# Association of amyloid and cardiovascular risk with cognition: Findings from KBASE

**DOI:** 10.1002/alz.14290

**Published:** 2024-11-07

**Authors:** Soumilee Chaudhuri, Desarae A. Dempsey, Yen‐Ning Huang, Tamina Park, Sha Cao, Evgeny J. Chumin, Hannah Craft, Paul K. Crane, Shubhabrata Mukherjee, Seo‐Eun Choi, Phoebe Scollard, Michael Lee, Connie Nakano, Jesse Mez, Emily H. Trittschuh, Brandon S. Klinedinst, Timothy J. Hohman, Jun‐Young Lee, Koung Mi Kang, Chul‐Ho Sohn, Yu Kyeong Kim, Dahyun Yi, Min Soo Byun, Shannon L. Risacher, Kwangsik Nho, Andrew J. Saykin, Dong Young Lee, for the KBASE Research Group

**Affiliations:** ^1^ Center for Neuroimaging Department of Radiology and Imaging Sciences Indiana University School of Medicine Indianapolis Indiana USA; ^2^ Indiana Alzheimer's Disease Research Center Indiana University School of Medicine Indianapolis Indiana USA; ^3^ Medical Neuroscience Graduate Program Stark Neurosciences Research Institute Indiana University School of Medicine Indianapolis Indiana USA; ^4^ Department of Biostatistics and Health Data Science Indiana University School of Medicine Indianapolis Indiana USA; ^5^ Department of Medicine University of Washington Seattle Washington USA; ^6^ Department of Neurology Boston University Boston Massachusetts USA; ^7^ Department of Psychiatry and Behavioral Sciences University of Washington Seattle Washington USA; ^8^ Geriatrics Research, Education, and Clinical Center VA Puget Sound Health Care System Seattle Washington USA; ^9^ Department of General Internal Medicine Harborview Medical Center University of Washington School of Medicine Seattle Washington USA; ^10^ Vanderbilt Memory & Alzheimer's Center Vanderbilt University Medical Center Nashville Tennessee USA; ^11^ Department of Neuropsychiatry SMGSNU Boramae Medical Center Dongjak‐gu Seoul Republic of Korea; ^12^ Department of Radiology Seoul National University Hospital Jongno‐gu Seoul Republic of Korea; ^13^ Department of Nuclear Medicine SMGSNU Boramae Medical Center Dongjak‐gu Seoul Republic of Korea; ^14^ Institute of Human Behavioral Medicine Medical Research Center Seoul National University Jongno‐gu Seoul Republic of Korea; ^15^ Department of Neuropsychiatry Seoul National University Hospital Jongno‐gu Seoul Republic of Korea; ^16^ Department of Psychiatry Seoul National University College of Medicine Jongno‐gu Seoul Republic of Korea; ^17^ School of Informatics and Computing Indiana University Indianapolis Indiana USA; ^18^ Department of Medical and Molecular Genetics Medical Research and Library Building Indiana University School of Medicine Indianapolis Indiana USA

**Keywords:** Alzheimer's disease, amyloid, CN, cognition, Framingham Risk Score, Korean older adults, longitudinal, MCI, vascular risk factors

## Abstract

**BACKGROUND:**

Limited research has explored the effect of cardiovascular risk and amyloid interplay on cognitive decline in East Asians.

**METHODS:**

Vascular burden was quantified using Framingham's General Cardiovascular Risk Score (FRS) in 526 Korean Brain Aging Study (KBASE) participants. Cognitive differences in groups stratified by FRS and amyloid positivity were assessed at baseline and longitudinally.

**RESULTS:**

Baseline analyses revealed that amyloid‐negative (Aβ–) cognitively normal (CN) individuals with high FRS had lower cognition compared to Aβ– CN individuals with low FRS (*p* < 0.0001). Longitudinally, amyloid pathology predominantly drove cognitive decline, while FRS alone had negligible effects on cognition in CN and mild cognitive impairment (MCI) groups.

**CONCLUSION:**

Our findings indicate that managing vascular risk may be crucial in preserving cognition in Aβ– individuals early on and before the clinical manifestation of dementia. Within the CN and MCI groups, irrespective of FRS status, amyloid‐positive individuals had worse cognitive performance than Aβ– individuals.

**Highlights:**

- Vascular risk significantly affects cognition in amyloid‐negative older Koreans.
- Amyloid‐negative CN older adults with high vascular risk had lower baseline cognition.
- Amyloid pathology drives cognitive decline in CN and MCI, regardless of vascular risk.
- The study underscores the impact of vascular health on the AD disease spectrum.

## 1 BACKGROUND

Prevention of Alzheimer's disease (AD) by targeting modifiable vascular risk factors has been a focal point of study in the absence of an effective and safe treatment strategy.^1^, ^2^, ^3^ Both the American Heart Association (AHA) and Lancet Committee have identified lifestyle (smoking, diet, etc.) and biological (blood pressure, total cholesterol [TC], etc.) metrics conducive to vascular health and dementia prevention.^4^, ^5^ While these vascular risk factors have been associated with cognitive decline, individually and through systemic cardiovascular measures such as the Framingham General Cardiovascular Risk Score (FRS),^6^, ^7^, ^8^ most of these studies have predominantly focused on non‐Hispanic white (NHW) populations in Western countries.^3^, ^9^, ^10^, ^11^, ^12^, ^13^, ^14^, ^15^, ^16^, ^17^

By 2030, dementia is projected to increase by 107% in Asia, with high regional variation in dementia and cardiovascular disease (CVD) burden in different East Asia countries.^18^, ^19^, ^20^, ^21^ South Korea has one of the most rapidly aging populations and a higher prevalence of dementia and CVD burden than most Asian and Western countries.^22^, ^23^, ^24^, ^25^ Several studies within this region have noted a high incidence of CV risk being associated with cognitive decline and increased risk for AD.^26^, ^27^, ^28^, ^29^, ^30^ However, these findings have been conflicting and primarily focused on singular CV risk factors that influence dementia in Koreans.^31^, ^32^, ^33^, ^34^, ^35^, ^36^ Few studies in this region have investigated the relationship between vascular risk and cognition and their interplay with amyloid, a pathophysiological hallmark of AD.

Within North America, while some studies have demonstrated a positive correlation between CV risk and cognitive decline in East Asian subgroups,^30^, ^32^, ^37^, ^38^, ^39^, ^40^, ^41^, ^42^ these findings have been inconsistent^3^, ^10^, ^11^, ^13^, ^43^ as well. Thus, there is a significant gap in understanding how CV risk affects cognition based on amyloid pathology in the AD spectrum among older Korean adults due to the limited number of longitudinal dementia studies focusing specifically on East Asian or East Asian American populations. Understanding the relationship between vascular risk, amyloid pathology, and cognition in specific East Asian subgroups is crucial due to variations in the prevalence and impact of modifiable vascular risk factors as well as racial and ethnic differences in amyloid pathology presentation among these groups.^44^, ^45^, ^46^, ^47^

Recognizing these needs, we leveraged the Korean Brain Aging Study for the Early Diagnosis and Prediction of Alzheimer's Disease (KBASE) data to focus on understanding vascular risk and cognition in a specific Asian subgroup: older Korean adults. KBASE is an initiative modeled after the Alzheimer's Disease Neuroimaging Initiative (ADNI),^48^ and this particular study utilized cardiometabolic variables and the amyloid status of participants in KBASE over 4 years. This study could inform further iterations of ADNI as well as future clinical practice, particularly in the realm of intervention strategies tailored to East Asian American diasporas. Moreover, this study is crucial as it provides (1) valuable insights into the broader East Asian context, as well as (2) significant implications for Asian American subgroups in North America regarding the association of CV risk and amyloid with longitudinal cognitive decline.

Therefore, our overall goal was to understand how vascular risk and amyloid pathology influence cognitive decline among older Korean adults. Specifically, vascular burden was quantified using the FRS, and participants were categorized into four groups based on combinations of FRS (FRS high or FRS low with a median split) and amyloid status (amyloid beta Aβ+ or Aβ– based on a cutoff of 1.24 standardized uptake value ratio [SUVR]). Cognitive function was evaluated using standardized neuropsychological tests processed with structural equation models to produce domain scores for memory, executive functioning, language, and visuospatial function. Analysis of variance (ANOVA) was employed at baseline to analyze cognitive differences among these groups within three clinical diagnosis groups. Longitudinal mixed‐effects models spanning 4 years from the initial visit captured cognitive changes within these groups. We hypothesized that higher CV risk, as measured by the FRS, will be associated with greater amyloid burden and poorer cognitive status over time, with these associations varying by clinical diagnosis.

## 2 METHODS

### 2.1 Study population

The data presented in this article were collected as part of the first iteration of the KBASE, an ongoing prospective study that began in 2014. The KBASE study protocol was approved by the Institutional Review Boards of Seoul National University Hospital and Seoul Metropolitan Government‐Seoul National University (SMG‐SNU) Boramae Center (Seoul, South Korea) and conducted in accordance with the Declaration of Helsinki. A total of 526 individuals (cognitively normal [CN] = 286, mild cognitive impairment [MCI] = 148, and AD = 92), who participated in the KBASE study between 2014 and 2018 were included. These participants had complete baseline information for amyloid positron emission tomography (PET) imaging, cognitive tests, and cardiometabolic variables such as body mass index (BMI), TC, blood pressure (BP), and so forth. Participants' ages ranged from 55 to 90 years. Young CN individuals, below age 55, were excluded from the study sample for analysis but were used for determination of amyloid positivity.

**RESEARCH IN CONTEXT**

1. **Systematic review**: The authors reviewed the literature using traditional sources like PubMed and found limited publications investigating the relationship between cardiovascular risk and amyloid pathology in Alzheimer's disease (AD) in East Asians.
2. **Interpretation**: Our study revealed distinct cognitive effects based on vascular risk and amyloid status in older Korean adults. In cognitively normal (CN) individuals, those with high vascular risk and low amyloid had worse cognition than those with low vascular risk and low amyloid. Longitudinally, amyloid pathology predominantly drove cognitive decline, while vascular risk alone or with amyloid had negligible effects on cognition.
3. **Future directions**: Our study enhances the understanding of vascular risk in AD progression in a diverse East Asian population with low vascular burden. The differential role of vascular risk and amyloid pathology in cognition across the AD spectrum warrants attention. Additionally, exploring associations of amyloid and vascular risk in pre‐symptomatic stages could help identify early intervention targets.

**FIGURE 1:**
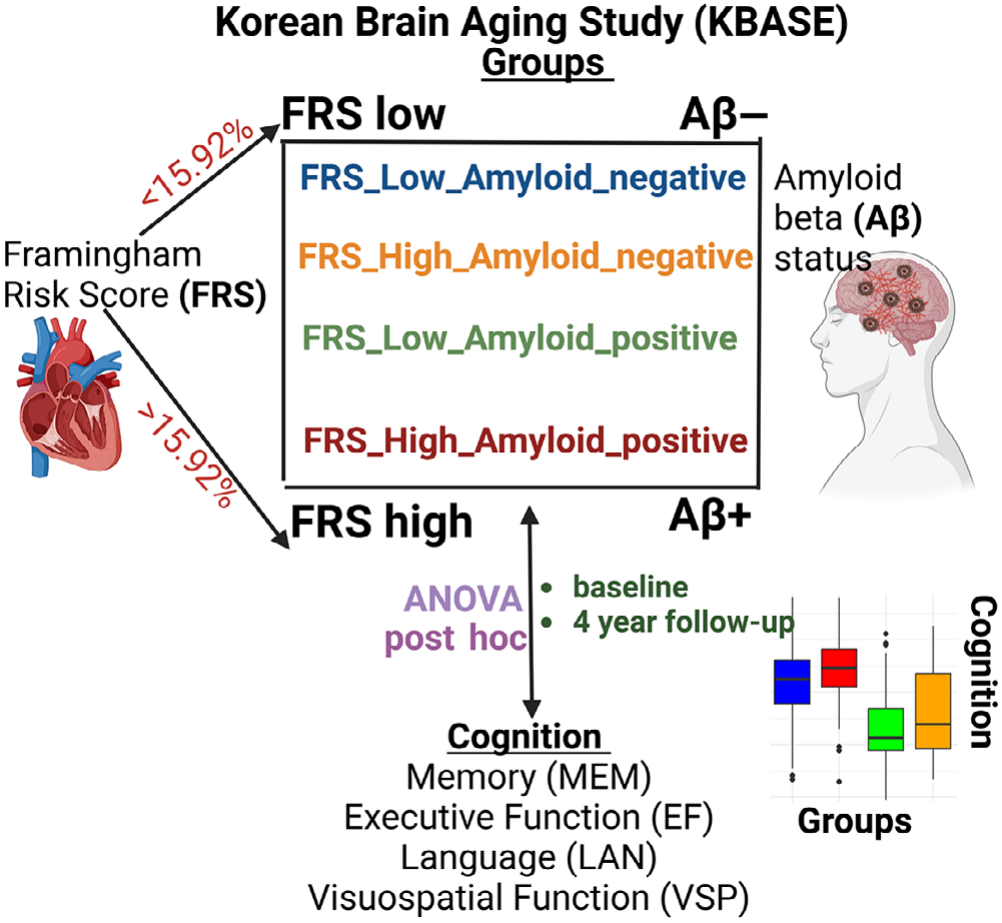
Overall study design. Vascular burden was quantified using the Framingham General Cardiovascular Risk Score (FRS), and participants were categorized into four groups based on combinations of FRS (FRS high or FRS low with a median split) and amyloid status (Aβ+ or Aβ– based on a cutoff of 1.2373). Cognitive function was evaluated using standardized neuropsychological tests processed with structural equation models to produce domain scores for memory, executive functioning, language, and visuospatial. Analysis of variance was employed at baseline to analyze cognitive differences among these groups and within each clinical diagnosis. Longitudinal mixed‐effects models spanning 4 years from the initial visit captured cognitive changes within these groups. Aβ, amyloid beta; ANOVA, analysis of variance; EXF, composite score for executive functioning; FRS, Framingham General Cardiovascular Risk Score; KBASE, Korean Brain Aging Study for the Early Diagnosis and Prediction of Alzheimer's Disease; LAN, composite score for language; MEM, composite score for memory, VSP, composite score for visuospatial functioning.

The CN participants had no diagnosis of MCI or dementia and a Clinical Dementia Rating (CDR) score of 0. Participants with MCI had a global CDR of 0.5 and fulfilled the core clinical criteria for diagnosis of MCI according to the recommendations of the National Institute on Aging–Alzheimer's Association guidelines (NIA‐AA).^49^ Participants with AD dementia had a global CDR score of 0.5 or 1 and met the criteria for dementia in accordance with the Diagnostic and Statistical Manual of Mental Disorders, 4th edition, text revision (DSM‐IV‐TR), and the criteria for probable AD dementia in accordance with the NIA‐AA.^50^ The exclusion criteria were (1) the presence of any psychiatric or neurological disorders that could affect mental function, (2) severe communication problems that would make assessments or brain scans difficult, (3) contraindications for magnetic resonance imaging (MRI) scanning, (4) absence of a reliable informant, or (5) being illiterate. Details of the KBASE cohorts and recruitment and exclusion criteria have previously been described.^51^ Demographic characteristics (age, sex, and education) were from self‐reports. A visual summary of our study methods is presented in Figure 1.

### 2.2 Cognitive function assessments

As previously described, participants underwent comprehensive neuropsychological testing, following a standardized protocol incorporating the Consortium to Establish a Registry for Alzheimer's Disease, Korean version (CERAD‐K) neuropsychological battery.^51^, ^52^ Cognitive composite scores were created and harmonized using the same workflow as previously described.^53^ Briefly, items administered were categorized into memory, executive functioning, visuospatial function, language, or none of these domains. Investigators ensured identical scoring of anchor items to previous English‐based studies. Anchor items were those identified as having been administered and scored identically in the Korean and English‐based test versions. We generated cognitive scores that were on the same scale, enabling comparisons across domains as well as other Alzheimer's study cohorts such as the ADNI. Full details pertaining to the harmonization and co‐calibration scoring and analysis for KBASE can be found in the Supplementary Materials.

This study focused on key cognitive function metrics: baseline cognitive function and longitudinal trajectory of cognitive decline. Baseline cognitive function was determined using the harmonized scores from participants’ initial cognitive assessments. These baseline cognition scores, pertaining to each of the four cognitive domains, were subsequently used as endophenotypes in our analyses.

### 2.3 CV risk factors

The presence of vascular risk factors (VRFs), including diabetes (Diab), hypertension (HTN), dyslipidemia (HLD), coronary artery disease (CAD), transient ischemic attack (TIA), and stroke, was assessed from data collected during systematic interviews by trained nurses with participants and their informants. Smoking status (never/former/smoker) was evaluated through interviews with nurses. BMI was calculated as weight in kilograms divided by the square of the height in meters. It was measured at the baseline visit. Trained research nurses measured the participants’ height and body weight using standard anthropometric methods.

Vascular burden was quantified using the FRS. The FRS is an aggregated sex‐specific measure of CV burden constructed based on age, TC, high‐density lipoprotein (HDL) cholesterol, systolic and diastolic pressures, smoking, and diabetes.^54^, ^55^ The FRS, defined as the risk of having a 10‐year risk of coronary heart disease (CAD), has been reported to be associated with cognitive decline and brain pathology; it is a widely reported standardized measure of systemic vascular risk.^56^ Several meta‐analyses have validated the FRS in multiethnic populations, finding that the FRS works well in populations with Asian ancestry.^57^, ^58^ This version of the score has been validated and employed in numerous prior studies.^14^, ^15^, ^16^, ^17^, ^56^, ^59^, ^60^, ^61^ We had a median FRS of 15.92%, and participants below that threshold were characterized into the FRS low group for analyses. The specific variables used in the FRS are displayed and quantified in Table 1.

**TABLE 1:** Participant characteristics at baseline in mean (SD).

	Mean (SD)				
Characteristic	CN (*N* = 286)	MCI (*N* = 148)	AD (*N* = 92)	Total (*N* = 526)	*p*‐value^a^
Age, years	69.02 (8.05)	73.51 (6.91)	72.50 (7.69)	70.89 (7.94)	<0.0001
Age range, years	55–87	55–90	55–85	55–90	NA
Female no. (%)	48.60	34.46	31.52	41.63	<0.01
Education, years	11.92 (4.82)	10.24 (4.50)	9.59 (5.42)	11.04 (4.94)	<0.0001
Aβ positive (%)	4.21	35.13	68.48	24.19	<0.0001
*APOE ε*4 carriers (%)	18.53	39.86	57.61	31.37	<0.0001
Framingham General Cardiovascular Risk Score (FRS) variables					
FRS (%)	16.69 (10.13)	21.47 (12.27)	19.45 (10.99)	18.52 (11.10)	<0.001
Systolic blood pressure, mm Hg	125.63 (16.85)	124.68 (16.62)	128.61 (17.17)	123.75 (16.57)	0.74
Diabetes (%)	16.43	18.92	15.22	16.92	0.72
Current smoker (%)	6.99	2.70	4.35	5.32	0.15
Treatment with anti‐hypertensive medication (%)	44.75	52.70	38.04	45.82	0.07
HDL cholesterol	53.83 (14.23)	55.37 (12.98)	56.27 (13.00)	54.69 (13.69)	0.10
Total cholesterol	185.50 (35.05)	186.26 (40.63)	192.81 (44.57)	186.99 (38.48)	0.15
Other cardiometabolic variables					
BMI	24.21 (2.98)	24.76 (3.13)	23.82 (2.60)	24.30 (3.15)	0.73
LDL cholesterol	108.91 (29.77)	108.97 (37.81)	115.04 (41.50)	110.00 (34.43)	0.20
CAD (%)	5.24	4.05	5.43	4.94	0.84
Stroke (%)	0	0	0	0	NA
TIA (%)	0.70	0.67	0	0.57	0.72
PET‐amyloid (log‐transformed)					
Gl_Ctx_CL	0.16 (0.11)	0.30 (0.19)	0.46 (0.19)	0.25 (0.19)	<0.0001
Cognition composites					
MEM	0.50 (0.50)	−0.50 (0.46)	−1.14 (0.43)	−0.067 (0.81)	<0.0001
EXF	0.73 (0.61)	0.15 (0.53)	−0.28 (0.67)	0.39 (0.72)	<0.0001
VSP	0.33 (0.92)	−0.50 (1.10)	−1.35 (1.33)	−0.22 (1.23)	<0.0001
LAN	0.54 (0.62)	−0.04 (0.57)	−0.47 (0.64)	0.20 (0.73)	<0.0001
Follow‐up, years (for longitudinal analyses)	2.00 (1.414)	2.00 (1.415)	2.00 (1.415)	2.00 (1.41)	1.00

Abbreviations: AD, Alzheimer's disease; *APOE*, apolipoprotein E; Aβ, amyloid beta; BMI, body mass index; CAD, coronary artery disease; CN, cognitively normal; EXF, composite score for executive functioning; FRS, Framingham's General Cardiovascular Risk Score; Gl_Ctx_CL, global cortical Pittsburgh Compound B standardized uptake value ratio from PET scans; HDL, high‐density lipoprotein; LAN, composite score for language; LDL, low‐density lipoprotein; MCI, mild cognitive impairment; MEM, composite score for memory; PET, positron emission tomography; TIA, transient ischemic attack; VSP, composite score for visuospatial functioning.

^a^ Chi‐squared test for categorical variables and analysis of variance (ANOVA) tests for continuous variables.

### 2.4 Amyloid neuroimaging biomarkers

#### 2.4.1 Amyloid PET image acquisition

The details of amyloid PET image acquisition have been described previously.^51^ Participants underwent simultaneous 3D [11C] Pittsburgh Compound B [PiB]‐PET and 3D T1‐weighted MRI using the 3.0 T PET‐MR scanner. After intravenous administration of ∼555 MBq of [11C] PiB (range, 450 to 610 MBq), and a 40‐min uptake period, a 30‐min emission scan was obtained (4‐ to 5‐min frames). PiB‐PET data were collected in list mode and processed for routine corrections such as uniformity, ultrashort echo time (UTE)‐based attenuation, and decay corrections, and were reconstructed into a 256 × 256 image matrix using iterative methods (six iterations with 21 subsets).

#### 2.4.2 Amyloid PET image processing

PiB‐PET images for the KBASE cohort were preprocessed with Statistical Parametric Mapping 12 (SPM12); https://www.fil.ion.ucl.ac.uk/spm/software/spm12/). First, 40‐ to 70‐min static PiB‐PET images were created with motion correction between frames. Each participant's static PiB‐PET images were co‐registered with each individual's T1 structural image from the same visit. Next, voxel‐based segmentation of the T1 images generated transformation matrices to normalize each T1 image to standard Montreal Neurological Institute (MNI) space. The transformation matrices were then used to normalize the aligned static PiB‐PET images to MNI space. Finally, normalized PiB‐PET scans were intensity normalized to create SUVR images, using a cerebellar grey matter region of interest (ROI) from the Centiloid project (https://www.gaain.org/centiloid‐project)^62^; and smoothed using an 8‐mm full‐width half maximum (FWHM) kernel. For this study, we used the global cortical amyloid SUVR measure, which was log‐transformed to reduce skewness.

### 2.5 Blood testing and laboratory assessments

Overnight fasting blood samples were collected from each participant. Laboratory tests including serum lipids (TC, HDL cholesterol, low‐density lipoprotein [LDL] cholesterol, and triglycerides) were measured. Genomic DNA was isolated from whole blood, followed by apolipoprotein E (*APOE*) genotyping using methods described previously.^51^, ^63^ Participants were grouped into one of two *APOE* groups, based on the absence or presence of at least one *ε*4 allele.

### 2.6 Statistical analyses

For demographic tables, continuous data were analyzed using ANOVA, and categorical data were analyzed using chi‐square tests. Descriptive statistics for participant characteristics, imaging biomarker levels, and cognitive performance were compared across diagnosis groups (CN, MCI, and AD) (Table 1) and by FRS and amyloid positivity groups (Table 2).

**TABLE 2:** Baseline participants' characteristics by FRS risk and amyloid status.

	Amyloid‐beta negative Aβ–		Amyloid‐beta positive Aβ+		
FRS category (median cutoff: 15.92)	FRS low (<15.92)	FRS high (>15.92)	FRS low (<15.92)	FRS high (>15.92)	*p*‐value^a^
*N*	176	147	86	115	
Diagnoses group					
CN, *n* (%)	142 (80.68)	99 (67.35)	27 (31.39)	17 (14.78)	<0.0001
MCI, *n* (%)	29 (16.48)	37 (25.17)	26 (30.23)	56 (48.69)	<0.01
AD, *n* (%)	5 (2.84)	11 (7.48)	33 (38.37)	42 (36.52)	<0.0001
Age, years	66.57 (8.23)	73.57 (6.68)	70.44 (8.04)	74.44 (5.40)	<0.0001
Age range, years	55–90	56–87	55–86	56–88	NA
Female no. (%)	63.07	20.41	66.28	17.39	
Education, years	12.19 (4.88)	9.86 (4.95)	12.21 (4.57)	9.94 (4.73)	<0.0001
*APOE ε*4 carriers (%)	13.64	16.33	50.00	63.48	< 0.0001
Cardiometabolic variables					
Systolic blood pressure, mm Hg	119.35 (15.50)	113.84 (15.47)	115.46 (14.30)	132.35 (14.40)	<0.0001
BMI	23.98 (2.97)	24.97 (2.25)	23.10 (2.91)	24.79 (3.05)	<0.0001
Diabetes (%)	10.79	28.57	8.14	18.26	<0.0001
Current smoker (%)	7.39	5.44	3.49	3.48	0.42
Treatment with anti‐hypertensive medication (%)	31.25	69.38	31.39	48.69	<0.0001
HDL cholesterol	55.42 (16.24)	53.22 (11.75)	55.17 (13.15)	54.96 (11.99)	0.50
LDL cholesterol	102.58 (29.14)	112.81 (34.39)	108.39 (32.73)	117.43 (41.34)	<0.01
Total cholesterol	181.66 (34.90)	189.79 (37.14)	181.69 (39.39)	195.47 (43.25)	<0.01
PET‐amyloid (log‐transformed)					
Gl_Ctx_CL	0.118 (0.04)	0.123 (0.04)	0.445 (0.13)	0.471 (0.15)	<0.0001

Abbreviations: AD, Alzheimer's disease; *APOE*, apolipoprotein E; Aβ, amyloid beta; BMI, body mass index; CN, cognitively normal; FRS, Framingham's General Cardiovascular Risk Score; Gl_Ctx_CL, global cortical Pittsburgh Compound B standardized uptake value ratio from PET scans; HDL, high‐density lipoprotein; LDL, low‐density lipoprotein; MCI, mild cognitive impairment; PET, positron emission tomography.

^a^ Chi‐squared test for categorical variables and analysis of variance (ANOVA) tests for continuous variables.

#### 2.6.1 Cross‐sectional analyses

At baseline, linear regression models were used to analyze the associations between (1) FRS and amyloid burden, (2) FRS and cognition, and (3) amyloid and cognition within each clinical diagnostic group. Additionally, to determine if FRS and amyloid imaging biomarkers interact with cognition at baseline, we estimated the association of FRS with each composite cognitive score using multivariable‐adjusted linear regression (composite cognitive score ∼ FRS × Amyloid + covariates). Covariates included baseline age, sex, *APOE* genotype, and educational attainment. Before analysis, all continuous variables were *z*‐transformed.

To further explore the interactive relationships between amyloid and FRS variables, we created four groups: FRS+ Aβ–, FRS– Aβ–, FRS+ Aβ+, and FRS– Aβ+. The FRS score was split into high and low groups based on a median split of 15.92%. The amyloid positivity cutoff was determined using a receiver operator characteristic (ROC) curve using baseline SUVR from the global cortical ROI from the Centiloid project^64^ to classify young CN from AD patients. The maximal stratification cutoff was 1.24% with 83% sensitivity and 100% specificity. To test for significant differences in cognition across the groups based on FRS and amyloid status, we ran two‐way analyses of covariance (ANCOVAs) in each diagnostic subgroup (CN, MCI, and AD) separately.

The *p*‐value threshold was maintained at 0.05, and all obtained *p*‐values were multiplied by 4 to account for Bonferroni correction. This adjustment ensured that the overall type I error rate was controlled for the presence of four cognitive domains. For instance, if the raw *p*‐value was 0.0042, the adjusted *p*‐value presented would be 0.0168. All reported *p* values shown in our results are post‐Bonferroni correction, and the ones that survived the correction threshold have been designated with * (*p* value < 0.05), ** (*p* value < 0.01), *** (*p* value < 0.001), or **** (*p* value < 0.0001) for visibility. All statistical analyses were performed using R Studio, R version 4.3.3.

#### 2.6.2 Longitudinal analyses

At the 4‐year follow‐up, we used a linear mixed effects model (LMEM) to understand (1) how FRS and amyloid (categorical) associates with cognitive composite scores over time, (2) if FRS and amyloid interact synergistically together with time (FRS × Amyloid × Time) to predict cognitive decline, and (3) if FRS interacts with time (FRS × Time) independently alongside amyloid (Amyloid × Time) to predict cognitive decline. For each diagnosis group, these models were analyzed with longitudinal LMEMs that included random intercepts and slopes with unstructured covariance.^65^

First, linear mixed‐effects models were used to estimate *β*‐coefficients and significance levels for the associations between the four FRS and amyloid stratified groups (categorial) and annual change in four cognitive domains, with follow‐up time (in years) as the time scale. The fixed effect included age, sex, *APOE*, educational attainment, groups, time, and their interaction. The random effect model included random intercept and slope, allowing the individual differences at baseline and across follow‐up, defined as follows.

$$\begin{array}{ccc} & & \mathrm{lme}\;\left(\mathrm{Cognition}∼1+\mathrm{Age}+\mathrm{Sex}+\mathrm{APOEGrp}+\mathrm{Edu}\right.\\ & & \left.+\;\mathrm{Group}*\mathrm{time},\;\mathrm{random}=∼\mathrm{time}|\mathrm{id}\right) \end{array}$$

Then, we used the synergistic model to understand the three‐way combined effects of FRS and amyloid on cognitive decline over time (FRS × Amyloid × Time), whereas the additive model looked at the independent contributions of FRS and amyloid, with time on cognitive decline (FRS x Time and Amyloid x Time). For this, the model predictors included time, as well as baseline age, sex, *APOE* *ε*4 genotype, education (years), FRS, amyloid deposition, their interaction (cross‐product) with time, FRS × Amyloid interaction, and FRS × Amyloid × Time interaction.

The three‐way interactions examined the possible synergism between baseline FRS and amyloid deposition on cognitive trajectories. If this term was not significant, we ran reduced models that excluded this term (and the FRS × Amyloid interaction term) to examine the independent associations between FRS and amyloid with the rate of change in cognition (as indicated by the FRS × Time and Amyloid × Time interaction terms, respectively).

##### 2.6.2.1 Synergistic model

 

$$\begin{array}{ccc} & & \mathrm{lme}\left(\mathrm{Cognition}∼1+\mathrm{Age}+\mathrm{Sex}+\mathrm{APOEGrp}+\mathrm{Edu}\right.\\ & & \left.+\;\mathrm{FRS}*\mathrm{Amyloid}*\mathrm{time},\;\mathrm{random}=∼\mathrm{time}|\mathrm{id}\right) \end{array}$$

##### 2.6.2.2 Additive model

 

$$\begin{array}{ccc} & & \mathrm{lme}\left(\mathrm{Cognition}∼1+\mathrm{Age}+\mathrm{Sex}+\mathrm{APOEGrp}+\mathrm{Edu}\right.\\ & & \left.+\;\mathrm{FRS}*\mathrm{time}+\mathrm{Amyloid}*\mathrm{time},\mathrm{random}=∼\mathrm{time}|\mathrm{id}\right) \end{array}$$

These models were repeated for each diagnostic group (CN, MCI, and AD).

Model fit was assessed using goodness‐of‐fit statistics including the Bayesian Information Criterion (BIC). Likelihood ratio tests were conducted to determine the significance of predictor variables and interaction terms in explaining variance in cognitive performance across different diagnostic groups and time points. All continuous variables were *z*‐transformed beforehand. The *p*‐value threshold was maintained at 0.05, and all obtained *p*‐values were multiplied by 4 to account for Bonferroni correction. This adjustment ensured that the overall type I error rate controlled for the presence of four cognitive domains. For instance, if the raw *p*‐value was 0.0042, the adjusted *p*‐value presented would be 0.0168. All reported *p* values shown in our results are post Bonferroni correction, and the ones that survived correction threshold and have been designated with * (*p* value < 0.05), ** (*p* value < 0.01), *** (*p* value < 0.001), or **** (*p* value < 0.0001) for visibility. All statistical analyses were performed using R Studio, R version 4.3.3.

## 3 RESULTS

### 3.1 Participant demographics

Participant demographics are shown in Tables 1 and 2. Significant differences were observed across diagnostic groups in age, sex, education, amyloid positivity, and *APOE ε*4 carrier status (all *p* < 0.05; Table 1). FRS significantly differed across diagnostic groups (*p* < 0.001), and anti‐hypertension medications were marginally significant (*p* = 0.07). Systolic blood pressure, BMI, diabetes, current smoking status, HDL, LDL, TC levels, CAD, stroke, and TIA occurrence were not significantly different. As expected, significant differences were observed for amyloid deposition and cognition composite scores for cognitive domains (all *p* < 0.001; Table 1).

The prevalence of CN individuals differed significantly between the Aβ– and the Aβ+ groups (*p* < 0.0001), with a higher proportion of CN individuals observed in the Aβ– groups (Table 2). Conversely, the prevalence of MCI and AD significantly varied across the Aβ– and Aβ+ groups (*p* < 0.01, *p* < 0.0001, respectively), with higher proportions of MCI and AD individuals observed in the Aβ+ groups, particularly among those with FRS high (>15.92). Additionally, significant differences were noted in age (*p* < 0.0001), education level (*p* < 0.0001), *APOE ε*4 carrier status (*p* < 0.0001), cardiometabolic variables including systolic BP (*p* < 0.0001), BMI (*p* < 0.0001), diabetes prevalence (*p* < 0.0001), LDL cholesterol (*p* < 0.01), and total cholesterol (*p* < 0.01) across the four groups (Table 2).

### 3.2 Cross‐sectional analyses

*Association of cardiovascular risk burden with amyloid deposition and cognition composite scores for cognitive domains*.

We first examined the relationships between FRS, amyloid, and cognition through linear regression analyses within each diagnosis group. Continuous measures of FRS and amyloid were highly associated with each other only in the AD group (*β* = −0.068, *p* < 0.05) (Table S1, Figure S1). FRS alone was not significantly associated with any of the composite scores for cognitive domains within any of the clinical diagnosis groups (Tables 3 and S2‐S4). Amyloid alone was significantly associated (*β* = −1.005, *p* < 0.05) only with the memory domain of the MCI group (Tables 3 and S5‐S7). Similarly, the FRS and amyloid interaction (FRS × Amyloid) was significant only in the MCI group (*β* = 0.312, *p* < 0.05) for the memory domain (Tables 3 and S2‐S4). The effect of FRS also differed in amyloid‐positive and amyloid‐negative individuals in the MCI and AD groups (Figure S2); FRS seemed to have the biggest impact on amyloid‐negative individuals.

**TABLE 3:** *β* coefficients and associated significance levels for the multivariate‐adjusted linear regression models at baseline for the (1) association of FRS, (2) amyloid, and (3) FRS and amyloid, with changes in cognitive function in different domains for each diagnosis group.

*β* coefficients for the multivariate‐adjusted linear regression models for (1) association of FRS, (2) amyloid, and (3) FRS and amyloid, with changes in cognitive function at baseline					
Diagnosis	Predictors	MEM	EXF	VSP	LAN
CN	FRS	−0.051	−0.034	−0.154	0.431
	Amyloid	−0.229	−0.030	0.329	0.382
	FRS × Amyloid	0.103	0.155	0.606	0.180
MCI	FRS	−0.039	−0.008	−0.153	−0.012
	Amyloid	−1.005^***^	0.582	−0.655	−0.243
	FRS × Amyloid	0.312^*^	−0.025	0.051	0.064
AD	FRS	0.070	0.027	0.242	0.004
	Amyloid	−0.535	−0.341	−0.833	−0.470
	FRS × Amyloid	0.120	0.125	−0.382	0.117

*Note*: Covariates are age, sex, education, and *APOE ε*4 genotype. Detailed covariate statistics associated with each baseline model are included in Tables S2–S7.

Abbreviations: AD, Alzheimer's disease; *APOE*, apolipoprotein E; CN, cognitively normal; EXF, composite score for executive functioning; FRS, Framingham's General Cardiovascular Risk Score; LAN, composite score for language; MCI, mild cognitive impairment; MEM, composite score for memory; VSP, composite score for visuospatial functioning.

* *p*‐value < 0.05.

** *p*‐value < 0.01.

*** *p*‐value < 0.001.

**** *p*‐value < 0.0001.

**FIGURE 2:**
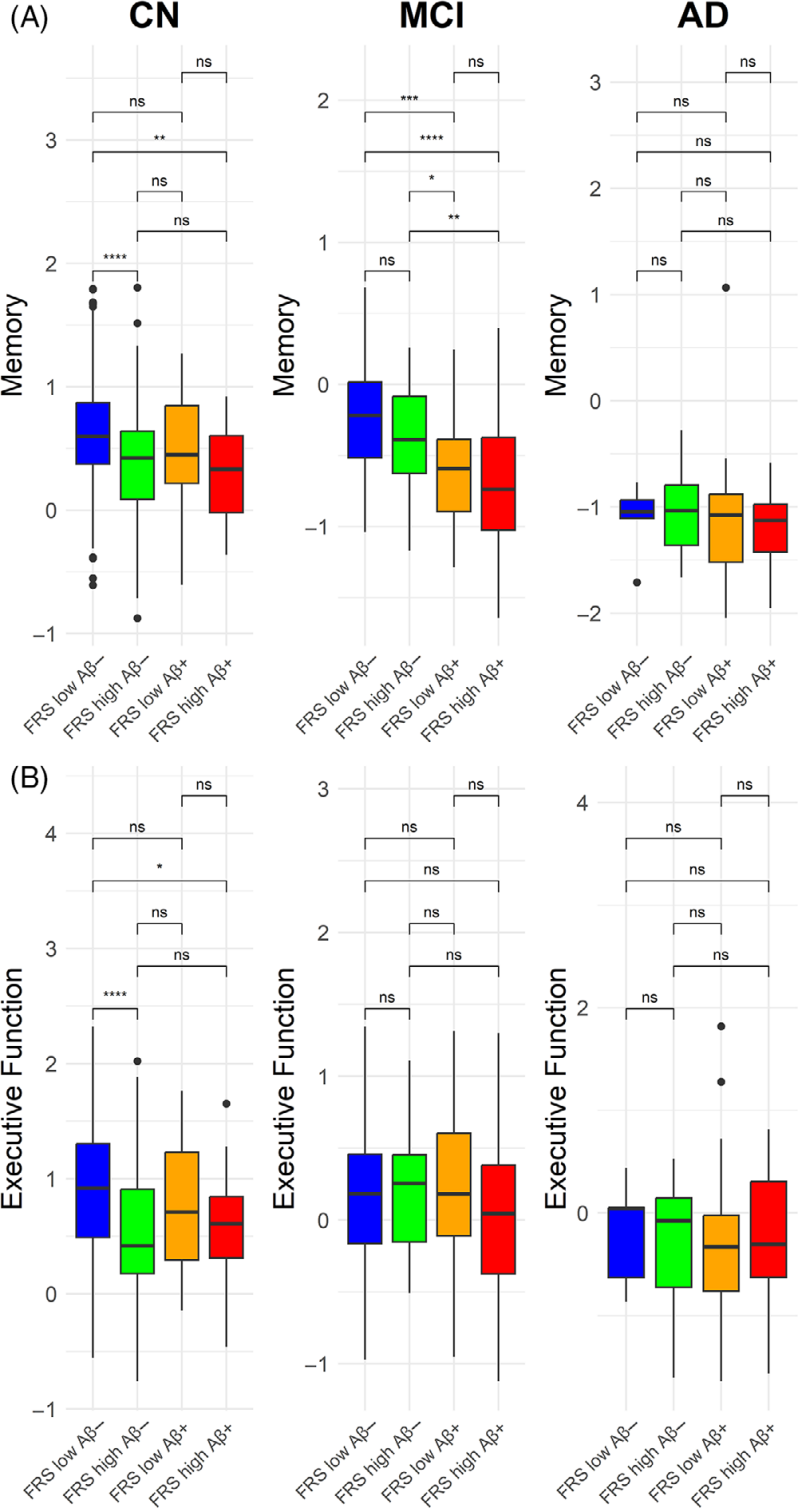
Analysis of covariance (ANCOVA) results for baseline cognitive differences in memory and executive function domains stratified by amyloid status and cardiovascular risk in each diagnosis group. (A) Baseline memory. Within each diagnosis, pairwise differences in baseline memory scores were assessed for the four groups stratified by amyloid status (Aβ–, Aβ+) and the Framingham General Cardiovascular Risk Score (FRS low, FRS high). Lower memory scores in FRS high Aβ– compared to FRS low Aβ– (*p* < 0.001) and FRS low Aβ+ individuals (*p* < 0.01) were found in the cognitively normal (CN) group. (B) Baseline executive function. Within each diagnosis, pairwise differences in baseline executive function were assessed for the four groups stratified by amyloid status (Aβ–, Aβ+) and FRS low versus FRS high. FRS high Aβ– individuals had lower executive function scores compared to FRS low Aβ– (*p* < 0.001) and FRS low Aβ+ individuals (*p* < 0.01) only in the CN group. AD, Alzheimer's disease; MCI, mild cognitive impairment. *p < 0.05, **p < 0.01, ***p< 0.001, ****p < 0.0001.

To better understand the relationship between FRS, amyloid, and cognition, we investigated cognitive differences among the four groups defined by amyloid positivity and FRS (FRS– Aβ–, FRS+ Aβ–, FRS– Aβ+, and FRS+ Aβ+). Pairwise comparisons revealed lower memory performance in FRS+ Aβ– individuals compared to FRS– Aβ– (*p* = 0.003) in the CN diagnosis group (Figure 2). This trend was present in all other cognitive domains for CN individuals but absent for the MCI and AD groups (Figures 2 and S3). Within the MCI group, cognitive differences were shown only in the memory domain primarily between FRS+ and FRS– individuals based on Aβ status (Figure 2). There were notable differences in memory performances between FRS– Aβ– and FRS– Aβ+ (*p* < 0.001) individuals as well as FRS+ Aβ– and FRS+ Aβ+ (*p* < 0.01) individuals within the MCI group.

### 3.3 Longitudinal analysis

*Assessment of synergistic or additive associations between cardiovascular risk factors and amyloid burden on longitudinal changes in cognition*.

**FIGURE 3:**
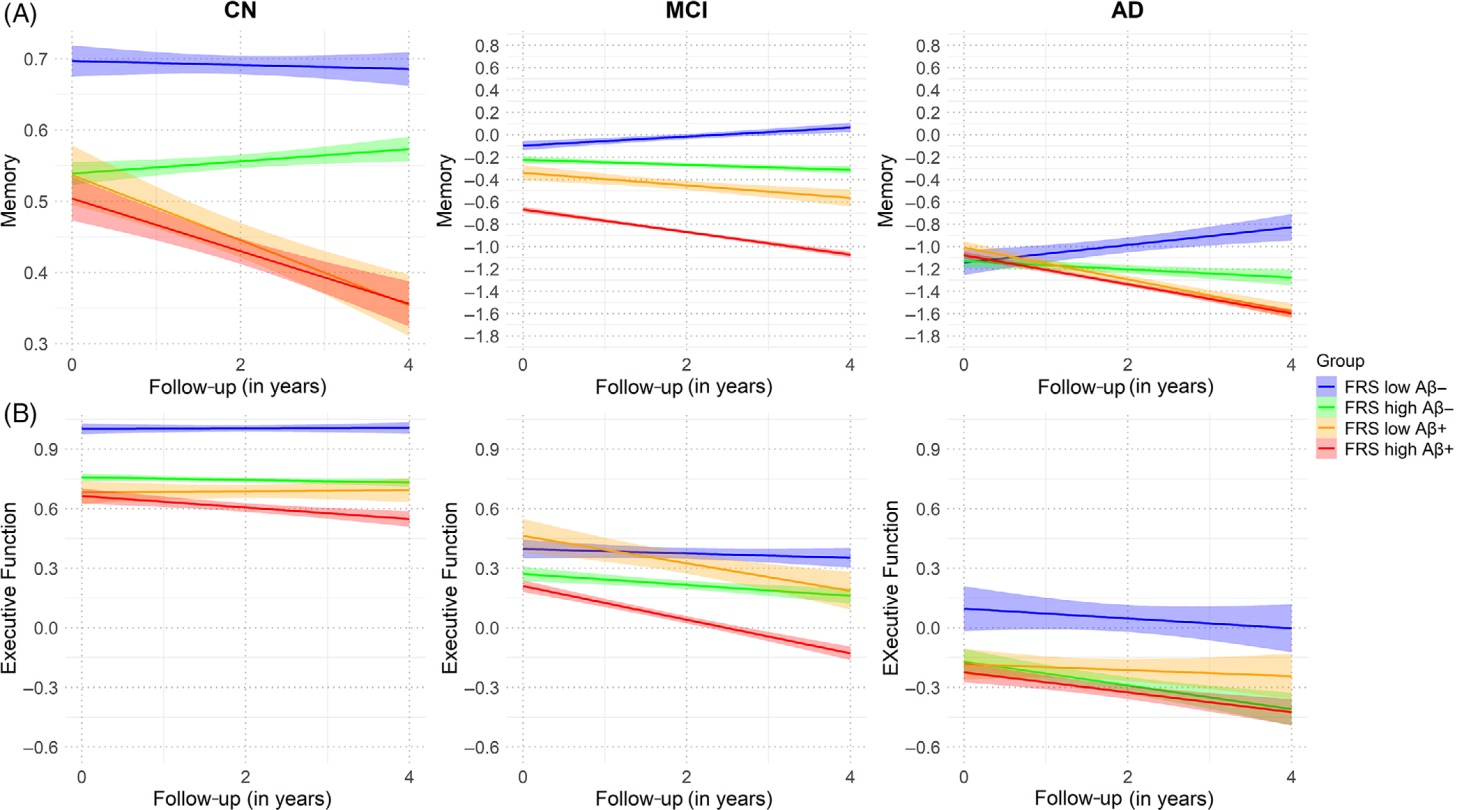
Longitudinal changes in (A) memory and (B) executive function stratified by amyloid status and cardiovascular risk groups within the clinical diagnosis groups of CN, MCI, and AD. These plots show the longitudinal changes in memory performance and executive functioning over a 4‐year follow‐up period. The participants in each diagnosis group were categorized into four groups based on their amyloid status (Aβ– or Aβ+) and the Framingham General Cardiovascular Risk Score (FRS low or FRS high). Distinct longitudinal patterns of cognitive decline for each clinical diagnosis group indicate that the impact of amyloid status and cardiovascular risk on memory and executive function differ depending on the clinical condition of the individuals. AD, Alzheimer's disease; CN, cognitively normal; MCI, mild cognitive impairment. *p < 0.05, **p < 0.01, ***p < 0.001, ****p < 0.0001.

Longitudinal analysis examining how cognitive composite scores change over a time of 4 years in CN and MCI groups showed that individuals with FRS+ Aβ– had slightly lower cognitive scores on average compared to the FRS– Aβ– group, but these associations were not statistically significant over the span of 4 years (Table 4, Figures 3 and S4).

**TABLE 4:** *β* coefficients and associated significance levels from linear mixed effects model for the association of FRS and amyloid groups (categorical) with longitudinal changes in domain‐specific cognitive function over follow‐up time, for each clinical diagnosis group.

Results from Linear Mixed Effects Model: *β* coefficients for the association of FRS and amyloid groups (categorical) with longitudinal changes in cognitive function in different domains over follow‐up time					
		Cognitive domains			
Diagnosis	FRS/Aβ categories × Time	MEM	EXF	VSP	LAN
CN	FRS low Aβ–* Time	Ref.	Ref.	Ref.	Ref.
	FRS high Aβ–* Time	0.000	0.000	0.000	−0.001
	FRS low Aβ+* Time	−0.059^**^	−0.021	−0.131^*^	−0.043^**^
	FRS high Aβ+* Time	−0.058^**^	−0.030	−0.132^*^	−0.041^**^
MCI	FRS low Aβ–* Time	Ref	Ref	Ref	Ref
	FRS high Aβ–* Time	−0.002	−0.001	−0.001	0.000
	FRS low Aβ+* Time	−0.076^**^	−0.104^*^	−0.210^**^	−0.107^**^
	FRS high Aβ+* Time	−0.078^**^	−0.102^*^	−0.210^**^	−0.107^**^
AD	FRS low Aβ–* Time	Ref.	Ref.	Ref.	Ref.
	FRS high Aβ–* Time	−0.003	0.002	0.000	0.003
	FRS low Aβ+* Time	0.003	−0.076	−0.164	−0.112
	FRS high Aβ+ * Time	0.008	−0.072	−0.162	−0.109

*Notes*: Covariates are age, sex, education, *APOE ε*4 genotype, time. Visual representation of categorical group differences within each diagnosis is shown in Figure 3 (MEM and EXF) and Figure S4 (VSP and LAN). Detailed covariate statistics associated with each longitudinal model are included in Tables S8‐S15.

Abbreviations: AD, Alzheimer's disease; Aβ, Amyloid beta; *APOE*, apolipoprotein E; CN, cognitively normal; EXF, composite score for executive functioning; FRS, Framingham's General Cardiovascular Risk Score; LAN, composite score for language; MCI, mild cognitive impairment; MEM, composite score for memory; Ref, reference group; VSP, composite score for visuospatial functioning.

* *p*‐value < 0.05.

** *p*‐value < 0.01.

*** *p*‐value < 0.001.

**** *p*‐value < 0.0001.

In the CN diagnosis group, the FRS– Aβ+ group exhibited significantly faster declines in memory (*β* = −0.059, *p* < 0.01), visuospatial function (*β* = −0.129, *p* < 0.05), and language (*β* = −0.054, *p* < 0.01) compared to the FRS– Aβ– group. The FRS+ Aβ+ group had a significantly steeper rate of decline compared to the FRS– Aβ– group (reference group) in all cognitive domains, except executive functioning (Table 4, Figures 3 and S4). This suggests that, for each year, compared to amyloid‐negative individuals with normal cognition and low Framingham scores, amyloid‐positive individuals with normal cognition and high Framingham scores experienced a steeper rate of decline in these cognitive domains.

Within the MCI diagnosis group, both the FRS– Aβ+ and FRS+ Aβ+ groups had a significantly steeper rate of decline compared to the reference group in all cognitive domains, indicating that, irrespective of vascular risk, individuals who are amyloid‐beta positive experience accelerated cognitive decline in the MCI stage. Within the AD diagnosis group, there were no differences in cognitive performance among these four groups (Table 4, Figures 3 and S4).

In assessing the synergistic (FRS × Amyloid × Time) interaction of FRS and amyloid on different cognitive domains over time, we found that the Amyloid × FRS × Time interaction was only significant for the visuospatial domain in the MCI diagnosis group (*β* = 0.327, *p* < 0.05). The impact of amyloid on cognitive decline (Amyloid × Time) was significant in the CN and MCI groups for most cognitive domains while there was no observed significant impact of FRS on cognitive decline (FRS × Time) in these diagnosis groups. However, the impact of amyloid on memory decline (Amyloid × Time) was significantly stronger than that of FRS (FRS × Time) in most cognitive domains in both the CN and MCI groups, as shown by the reported *β* values (Tables S8‐S11).

In assessing additive contributions of FRS and amyloid (FRS × Time and Amyloid × Time) on different cognitive domains over time, we found the only significant FRS × Time interaction was in the memory domain of the AD group (Table S12), but it was not accompanied by a significant Amyloid × Time interaction. Moreover, the effect of FRS with time (FRS × Time) was negligible on memory, language, executive function, and visuospatial functioning for the CN group; wherein lower memory was negligibly affected by FRS but significantly negatively affected by higher amyloid deposition. This pattern is repeated in the MCI group for several other cognitive domains where we see (1) a strong negative effect of amyloid (Amyloid × Time) and (2) a marginally (positive or negative) effect of FRS (FRS × Time) with time (Tables S12‐S15). We did not observe the aforementioned trends in FRS × Amyloid × Time (synergistic interaction) or Amyloid × Time or FRS × Time (additive interaction) within the AD group for any of the cognitive domains.

## 4 DISCUSSION

In a cross‐sectional and longitudinal analysis of 526 well‐characterized Korean older adults, we found that the interplay between CV risk factors, measured by the FRS and amyloid status, was associated with differences in cognitive trajectories based on clinical diagnoses. To understand how CV risk and amyloid interplay with cognitive trajectories of Korean older adults, we stratified the KBASE cohort into four groups based on the combination of amyloid status (Aβ– or Aβ+) and FRS categories (low [FRS–] or high [FRS+]). At baseline, in the CN subgroup, we found that individuals with FRS+ and Aβ– had worse cognitive performance on average compared to those with FRS– and Aβ–, in all domains of cognition. Within the MCI subgroup, cognitive differences were observed only between individuals with FRS+ Aβ– compared to those with FRS+ Aβ+, with the former group having higher cognitive scores on average. Interestingly, individuals with FRS– Aβ– within the CN group did not differ significantly from FRS+ Aβ– individuals in longitudinal cognitive performance. However, we did see a significant decline in the cognitive performance of Aβ+ individuals, irrespective of the FRS group, compared to Aβ– individuals in both the CN and MCI groups.

At baseline, a clear and significant difference in cognition between amyloid‐negative individuals with high FRS versus low FRS in the CN group points toward a differing role of CV health in conferring risk of cognitive decline, unexpectedly in the group with the least amyloid pathology. Previous studies in non‐demented Korean populations with no history of cerebrovascular diseases have found similar results wherein individuals with high CV risk had poorer cognitive function compared to those with lower vascular risk.^27^, ^32^, ^36^, ^66^ Using a CV model (Korean Risk Score) tailored for Koreans, Mun et al. found that higher CV risk was associated with poorer cognitive function among ∼8000 Korean older adults, with particularly strong effects in older women.^27^ Similarly, Cho et al. found that poorer CV health was associated with a high risk of dementia including AD and vascular dementia in ∼190,000 older Korean adults without prior dementia.^32^ However, none of these studies took into consideration the amyloid status of the participants. Our study findings are also consistent with what we see in North American cohorts, wherein Hohman et al.’s paper using ADNI and National Alzheimer's Coordinating Center (NACC) datasets found that the presence of amyloid pathology influences how the risk of stroke was associated with AD biomarkers. These findings were attributed to ADNI's enrollment protocol that ensures a restrictive vascular risk^8^ by excluding anyone with a high burden of cerebrovascular disease. As KBASE was modeled on ADNI and follows a similar protocol for enrollment, we also found this cohort to have a substantially low vascular burden at baseline with a median FRS of only 15.92% (Table 2). This is a study limitation that could explain why FRS influences cognitive decline when there is a low burden of amyloid (in CN), whereas in MCI individuals we see that the cognitive difference at baseline is mostly driven by amyloid pathology. Our baseline findings provide clues to the ongoing debate on whether vascular risk and amyloid are independently associated with cognitive impairment and ultimately suggest that controlling CV health might be important for individuals at low risk for AD and before clinical onset.

Our results from longitudinal analyses are more complex. We observed that within both the CN and MCI groups, irrespective of vascular risk, individuals who were Aβ‐positive experienced an accelerated cognitive decline compared to amyloid‐negative individuals. However, when considering the joint impact of CV risk, amyloid, and time (synergistic interaction) or separate contributions of CV risk and amyloid over time (additive contributions), we observed that changes in CV risk over time (FRS × Time) were only significant for memory in individuals with Alzheimer's disease. Additionally, the negative effect of amyloid over time (Amyloid × Time) was consistently significant across cognition of all diagnosis groups, except AD individuals. While similar longitudinal modeling of CV risk, amyloid, and cognition in Korean populations is lacking, most previous studies^67^, ^68^, ^69^ in North America have indicated additive and independent contributions of FRS and amyloid on cognition or their synergistic behavior^12^ in cognitively unimpaired individuals. However, our present longitudinal results in KBASE do not completely align with that.

Some limitations of this study warrant discussion. First, all versions of the metric of CV burden used for this study, the FRS, are known to inflate CV disease burden.^57^, ^70^, ^71^ Large population‐based studies that validate the use of the FRS in Asian and Asian American individuals are lacking, and it is possible that we overestimated the CV risk factor burden in these individuals. Population characteristics, data collection types, and the exclusion criteria of KBASE limited our ability to calculate other versions of the FRS, such as the Framingham Stroke Risk Profile (FSRP). Our study only had longitudinal data from three time points (baseline, Visit 1, Visit 2) over 4 years. We also did not examine the use of hypertensive and statin medications that have been shown to moderate the relationship.^72^, ^73^ Third, this study was designed to assess the lifestyle behaviors at baseline and was not updated during the follow‐up due to concerns about previously reported reverse causal relationship between lifestyle changes and cognitive impairment as the population ages.^74^ Fourth, our estimates are based on observational data and do not imply certain causality. Due to genetic or sociocultural factors which may affect the interplay between CV risk factors, amyloid, and cognitive trajectories, our findings may not be generalizable to other Asian subgroups or Asian American diasporas living in the United States, as our cohort consisted of Korean older adults residing in the Republic of Korea. The KBASE study design incorporated structural MRI which we plan to utilize in the future, but the first iteration did not incorporate T2‐FLAIR MRI, a measure that has been classically used to understand if CV burden interacts with cerebrovascular pathology such as structural lesions and white matter hyperintensity in the AD cascade leading to cognitive decline. Critical questions remain regarding whether the relationship between CV burden and amyloid might contribute to the ethnic disparities in cognitive aging among different sub‐populations. More studies on racially and ethnically diverse populations are warranted, especially given the low rates of diagnoses of clinical AD in East Asian groups and Asian American Pacific Islanders (AAPI).

However, this study is the first to highlight the differential impact of CV risk on cognition, depending on amyloid status and clinical diagnosis group in a Korean subgroup that was selected to have low vascular burden at baseline. This underscores the importance of considering both CV risk factors and amyloid pathology early on in understanding clinical manifestation and cognitive decline in the AD spectrum, particularly in ethnically diverse populations. We need future studies focusing on specific East Asian and AAPI subgroups that delineate possible interactions between vascular risk and amyloid through vascular injury (cerebrovascular diseases), which are thereby hypothesized to promote cognitive decline in non‐demented elderly.

Both vascular and amyloid mechanisms of cognitive decline and dementia play important roles in the aging brain and are hypothesized to impact neurocognitive trajectories. Our study provides evidence (1) that high CV burden may be a risk for cognitive decline in amyloid‐negative CN participants early on, and (2) points toward a primary role of amyloid pathology driving the cognitive decline, longitudinally. As we were also limited by a relatively small sample size, we also could not robustly confirm that FRS and amyloid, additively or synergistically, have a role in the development of dementia in this specific Asian cohort. Therefore, treatments for cognitive decline in Asian or AAPI cohorts should specifically consider both pathways, especially making note of CV burden in non‐demented elderly. Our findings further support the broad heterogeneity in dementia causation even within East Asian groups, emphasizing the critical need to target multiple pathways in ultimate efforts for prevention.

## CONFLICT OF INTEREST STATEMENT

Andrew J. Saykin has received support from Avid Radiopharmaceuticals, a subsidiary of Eli Lilly (in‐kind contribution of PET tracer precursor), and participated in Scientific Advisory Boards (Bayer Oncology, Eisai, Novo Nordisk, and Siemens Medical Solutions USA, Inc) and an Observational Study Monitoring Board (MESA, NIH NHLBI), as well as several other NIA External Advisory Committees. He also serves as Editor‐in‐Chief of *Brain Imaging and Behavior*, a Springer‐Nature journal. Other co‐authors have no competing interests in this study. Author disclosures are available in the supporting information.

## CONSENT STATEMENT

All human subjects provided informed consent.

## Supporting information



Supporting information

ICMJE Disclosure Form

## Data Availability

KBASE data will be included in future releases of the NIA ADSP harmonized phenotypic and genomic sequence data. The datasets used and/or analyzed in the present report are available from the corresponding author on reasonable request.
