# Supplementary material for: Association of amyloid and cardiovascular risk with cognition: Findings from KBASE
Source: Alzheimers Dement. 2024 Nov 7;20(12):8527–40. doi: 10.1002/alz.14290 (PMC11667546; doi:10.1002/alz.14290)
Supplement: Supplementary file 1 — Supporting information [file ALZ-20-8527-s001.docx]

**SUPPLEMENTARY FIGURES AND TABLES**


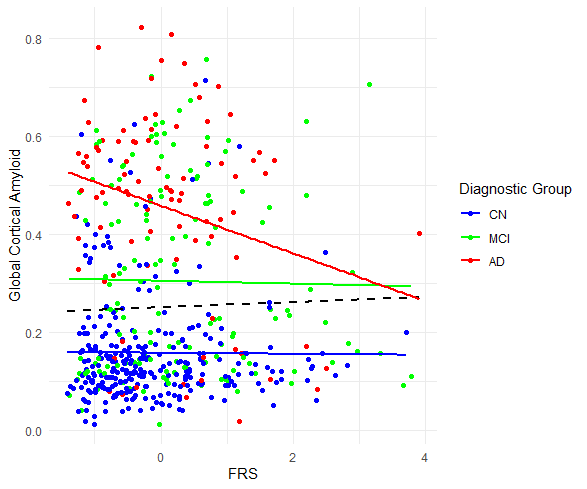


**Figure S1:** Cross-sectional linear regression results of the relationship between Framingham’s General Cardiovascular Risk Score (FRS) and global cortical amyloid as a continuous variable in the full sample (black dotted line), and stratified by diagnostic group, **Blue line:** Cognitive Normal (CN), **Green line:** Mild Cognitive Impairment (MCI), **Red line:** Alzheimer’s disease (AD)

Data is shown in Table 3 and Table S1.


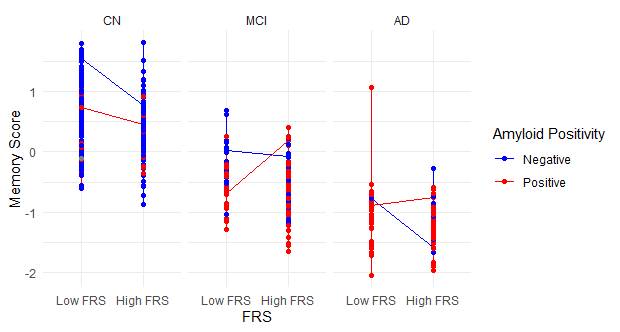


**Figure S2:** Cross-sectional interaction plots showing the relationship between low and high Framingham’s General Cardiovascular Risk Score (FRS) groups and amyloid positive and negative groups for the outcome of memory score, stratified by diagnostic group (CN = cognitive normal, MCI = mild cognitive impairment, AD = Alzheimer’s disease). The lines represent the mean FRS for each group.

The effect of FRS differs in amyloid positive and negative individuals in MCI and AD individuals, as shown by the interaction. Overall, FRS has the biggest impact on amyloid-negative individuals.


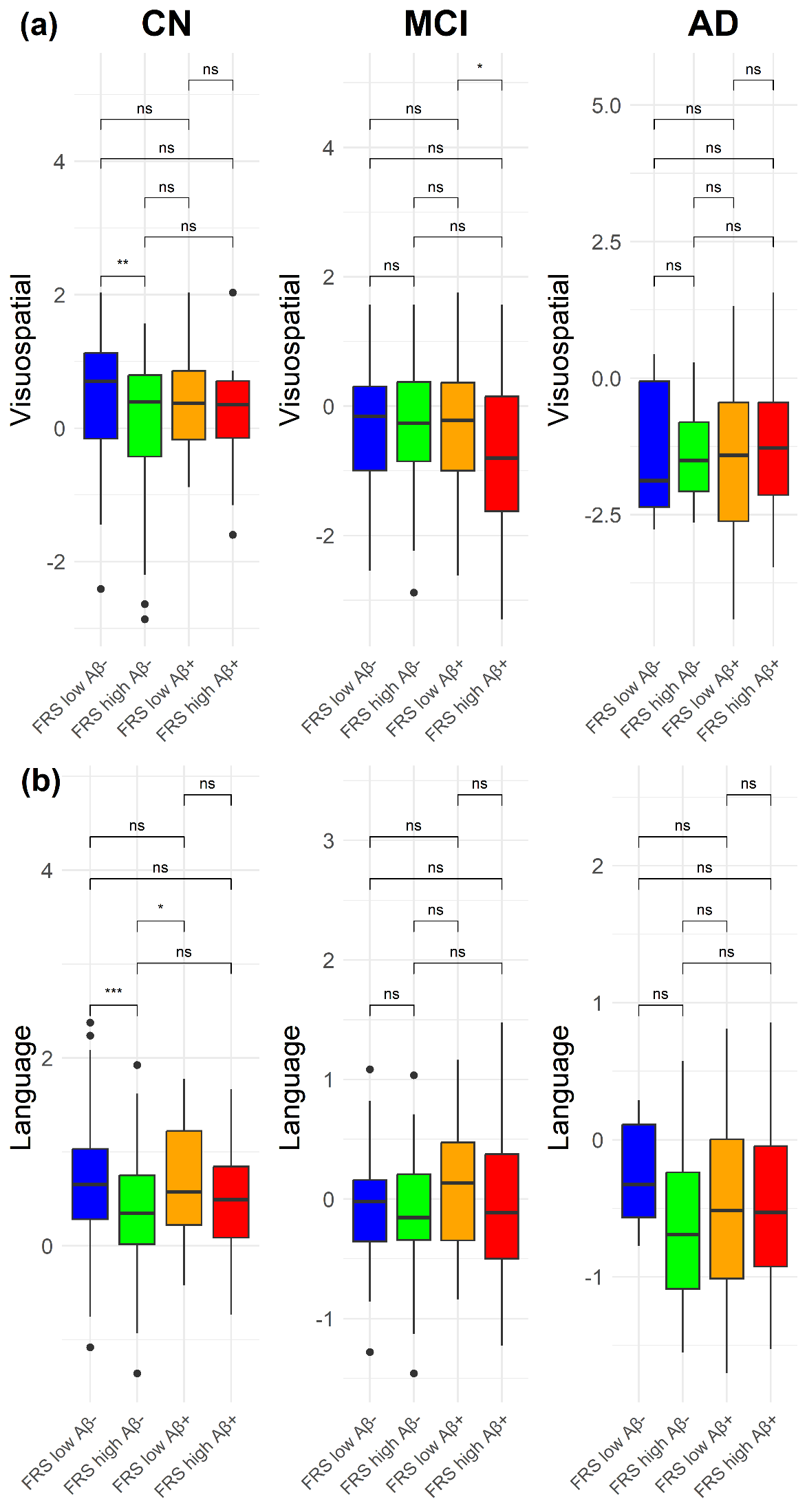


**Figure S3:** **Analysis of Covariance (ANCOVA) results for baseline cognitive differences in (a)visuospatial functioning and (b)language domains stratified by amyloid status and cardiovascular risk in each diagnosis.**

(a) **Baseline visuospatial functioning:** Within each diagnosis, pairwise differences in baseline visuospatial functioning scores were assessed for the four groups stratified by Amyloid status (Aβ-, Aβ+) and Framingham’s General Cardiovascular Risk Score (FRS low, FRS high). Lower scores in FRS high Aβ- compared to FRS low Aβ- (p < 0.01) were found in Cognitively Normal (CN) group.

(b) **Baseline language performance**: Within each diagnosis, pairwise differences in baseline language were assessed for the four groups stratified by Amyloid status (Aβ-, Aβ+) and Framingham’s General Cardiovascular Risk Score (FRS low, FRS high). FRS high Aβ- individuals had lower language performance scores compared to FRS low Aβ- (p < 0.001) individuals in Cognitively Normal (CN) group.

*p-value<0.05, **p-value<0.01, ***p-value<0.001, ****p-value<0.0001


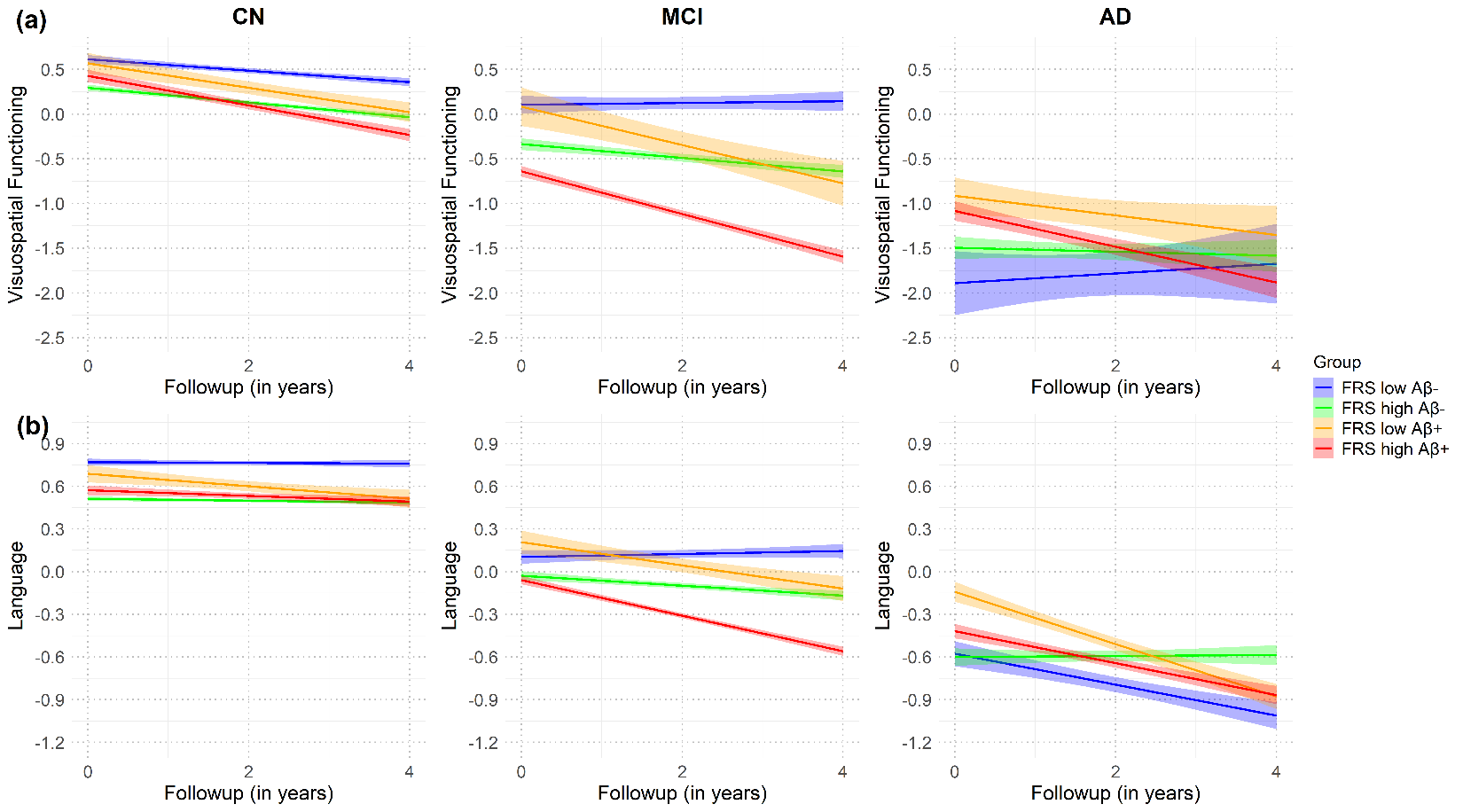


**Figure S4:** **Longitudinal changes in (a) visuospatial functioning and (b) language performance stratified by amyloid status and cardiovascular risk groups within CN, MCI, and AD.**

This plot shows the longitudinal changes in visuospatial functioning and language performance over a 4-year follow-up period. The participants in each diagnosis were categorized into four groups based on their Amyloid status (Aβ- or Aβ+) and Framingham’s General Cardiovascular Risk Score (FRS low or FRS high). **Blue line:** FRS low, Aβ-, **Green line:** FRS high, Aβ-, **Orange line:** FRS low, Aβ+, **Red line:** FRS high, Aβ+. **Left plot:** Cognitively Normal (CN) group, **Middle plot:** Mild Cognitive Impairment (MCI) group, **Right plot:** Alzheimer’s Disease (AD) group.

*p-value<0.05, **p-value<0.01, ***p-value<0.001, ****p-value<0.0001

| **Association of FRS and Amyloid within each diagnosis group**  **Model: Amyloid ~ FRS + covariates** | | | | | |
| --- | --- | --- | --- | --- | --- |
| **Diagnosis** | **PET-Amyloid** | **Predictors** | **Beta** | **SE** | **Adjusted p-value** |
| CN | Amyloid | FRS | -0.015 | 0.008 | 0.076 |
|  |  | Age | 0.004 | 0.001 | ****<0.0001 |
|  |  | Sex | 0.018 | 0.015 | 0.235 |
|  |  | APOE | 0.051 | 0.160 | **<0.01 |
|  | | | | | |
| MCI | Amyloid | FRS | -0.005 | 0.014 | 0.707 |
|  |  | Age | 0.001 | 0.001 | 0.612 |
|  |  | Sex | 0.031 | 0.034 | 0.360 |
|  |  | APOE | 0.201 | 0.027 | ****<0.0001 |
|  | | | | | |
| AD | Amyloid | FRS | -0.068 | 0.025 | *<0.05 |
|  |  | Age | -0.100 | 0.003 | 0.607 |
|  |  | Sex | 0.129 | 0.055 | *<0.05 |
|  |  | APOE | 0.107 | 0.038 | *<0.05 |

**Table S1:** Results of multivariable-adjusted association of FRS and amyloid, using linear regression (Covariates: age, sex, *APOE4* genotype) in CN, MCI and AD diagnosis groups. There was no observed interaction at the baseline of FRS and Amyloid to predict cognition in CN and MCI groups.

*p-value<0.05, **p-value<0.01, ***p-value<0.001, ****p-value<0.0001

| **Association of FRS and amyloid with baseline cognition in CN**  **Model: Cognition ~ FRS*Amyloid + covariates** | | | | |
| --- | --- | --- | --- | --- |
| **Cognition Composite** | **Model Predictor** | **Beta** | **Standard Error (SE)** | **Adjusted p-value** |
| MEM | FRS | -0.051 | 0.051 | 0.317 |
|  | Amyloid | -0.234 | 0.226 | 0.300 |
|  | FRS x Amyloid | 0.103 | 0.250 | 0.681 |
|  | Age | -0.020 | 0.003 | ****<0.0001 |
|  | Sex | 0.132 | 0.055 | *<0.05 |
|  | Education | 0.046 | 0.005 | ****<0.0001 |
|  | APOE | 0.147 | 0.061 | *<0.05 |
|  |  |  |  |  |
| EXF | FRS | -0.034 | 0.057 | 0.557 |
|  | Amyloid | -0.006 | 0.253 | 0.980 |
|  | FRS x Amyloid | 0.155 | 0.280 | 0.581 |
|  | Age | -0.035 | 0.004 | *<0.05 |
|  | Sex | 0.076 | 0.062 | 0.218 |
|  | Education | 0.057 | 0.006 | *<0.05 |
|  | APOE | -0.071 | 0.068 | 0.296 |
|  |  |  |  |  |
| VSP | FRS | -0.154 | 0.106 | 0.146 |
|  | Amyloid | 0.330 | 0.460 | 0.473 |
|  | FRS x Amyloid | 0.606 | 0.535 | 0.258 |
|  | Age | -0.026 | 0.007 | 0.0003 |
|  | Sex | 0.103 | 0.114 | 0.363 |
|  | Education | 0.096 | 0.011 | *<0.05 |
|  | APOE | -0.278 | 0.125 | 0.028 |
|  |  |  |  |  |
| LAN | FRS | 0.431 | 0.255 | 0.092 |
|  | Amyloid | -0.012 | 0.058 | 0.831 |
|  | FRS x Amyloid | 0.180 | 0.282 | 0.524 |
|  | Age | -0.030 | 0.004 | *<0.05 |
|  | Sex | 0.147 | 0.062 | 0.019 |
|  | Education | 0.069 | 0.006 | *<0.05 |
|  | APOE | 0.050 | 0.069 | 0.462 |

**Table S2:** Results of multivariable-adjusted association of FRS and amyloid at baseline to predict cognition, using linear regression (Covariates: age, sex, *APOE4* genotype, and education) in CN participants. There was no observed interaction at the baseline of FRS and Amyloid to predict cognition in CN. *p-value<0.05, **p-value<0.01, ***p-value<0.001, ****p-value<0.0001

| **Association of FRS and amyloid with baseline cognition in MCI**  **Model: Cognition ~ FRS*Amyloid + covariates** | | | | |
| --- | --- | --- | --- | --- |
| **Cognition Composite** | **Model Predictor** | **Beta** | **Standard Error (SE)** | **Adjusted p-value** |
| MEM | FRS | -0.039 | 0.052 | 0.455 |
|  | Amyloid | -1.072 | 0.202 | ****<0.0001 |
|  | FRS x Amyloid | 0.312 | 0.155 | *<0.05 |
|  | Age | -0.021 | 0.005 | ****<0.0001 |
|  | Sex | -0.064 | 0.080 | 0.42782 |
|  | Education | 0.024 | 0.008 | *<0.05 |
|  | APOE | 0.151 | 0.076 | *<0.05 |
|  |  |  |  |  |
| EXF | FRS | -0.008 | 0.067 | 0.904 |
|  | Amyloid | 0.579 | 0.261 | *<0.05 |
|  | FRS x Amyloid | -0.025 | 0.200 | 0.902 |
|  | Age | -0.006 | 0.007 | 0.327 |
|  | Sex | 0.176 | 0.103 | 0.091 |
|  | Education | 0.052 | 0.010 | *<0.05 |
|  | APOE | 0.062 | 0.098 | 0.530 |
|  |  |  |  |  |
| VSP | FRS | -0.153 | 0.131 | 0.247 |
|  | Amyloid | -0.704 | 0.517 | 0.175 |
|  | FRS x Amyloid | 0.051 | 0.393 | 0.898 |
|  | Age | 0.0003 | 0.013 | 0.982 |
|  | Sex | 0.112 | 0.206 | 0.587 |
|  | Education | 0.118 | 0.019 | *<0.05 |
|  | APOE | 0.300 | 0.192 | 0.119 |
|  |  |  |  |  |
| LAN | FRS | -0.012 | 0.068 | 0.860 |
|  | Amyloid | -0.258 | 0.265 | 0.332 |
|  | FRS x Amyloid | 0.064 | 0.203 | 0.751 |
|  | Age | -0.019 | 0.007 | *<0.05 |
|  | Sex | 0.146 | 0.105 | 0.166 |
|  | Education | 0.063 | 0.010 | *<0.05 |
|  | APOE | 0.119 | 0.099 | 0.232 |

**Table S3:** Results of multivariable-adjusted association of FRS and amyloid at baseline to predict cognition, using linear regression (Covariates: age, sex, *APOE4* genotype, and education) in MCI participants. Significant interaction was observed at the baseline of FRS and Amyloid to predict cognition in MCI only in the memory domain.

*p-value<0.05, **p-value<0.01, ***p-value<0.001, ****p-value<0.0001

| **Association of FRS and amyloid with baseline cognition in AD**  **Model: Cognition ~ FRS*Amyloid + covariates** | | | | |
| --- | --- | --- | --- | --- |
| **Cognition Composite** | **Model Predictor** | **Beta** | **Standard Error (SE)** | **Adjusted p-value** |
| MEM | FRS | 0.070 | 0.113 | 0.536 |
|  | Amyloid | -0.477 | 0.255 | 0.064 |
|  | FRS x Amyloid | 0.120 | 0.242 | 0.621 |
|  | Age | -0.003 | 0.006 | 0.639 |
|  | Sex | 0.022 | 0.130 | 0.865 |
|  | Education | 0.030 | 0.010 | *<0.05 |
|  | APOE | 0.128 | 0.091 | 0.165 |
|  |  |  |  |  |
| EXF | FRS | 0.027 | 0.185 | 0.885 |
|  | Amyloid | -0.335 | 0.419 | 0.427 |
|  | FRS x Amyloid | 0.125 | 0.398 | 0.754 |
|  | Age | 0.009 | 0.010 | 0.386 |
|  | Sex | 0.172 | 0.214 | 0.424 |
|  | Education | 0.038 | 0.016 | *<0.05 |
|  | APOE | 0.158 | 0.151 | 0.296 |
|  |  |  |  |  |
| VSP | FRS | 0.242 | 0.348 | 0.489 |
|  | Amyloid | -0.656 | 0.748 | 0.383 |
|  | FRS x Amyloid | -0.382 | 0.738 | 0.607 |
|  | Age | 0.035 | 0.019 | 0.060 |
|  | Sex | 0.403 | 0.395 | 0.311 |
|  | Education | 0.141 | 0.029 | ***<0.001 |
|  | APOE | 0.060 | 0.271 | 0.826 |
|  |  |  |  |  |
| LAN | FRS | 0.004 | 0.160 | 0.978 |
|  | Amyloid | -0.489 | 0.361 | 0.180 |
|  | FRS x Amyloid | 0.117 | 0.343 | 0.734 |
|  | Age | 0.008 | 0.009 | 0.331 |
|  | Sex | 0.193 | 0.185 | 0.298 |
|  | Education | 0.062 | 0.014 | ****<0.0001 |
|  | APOE | 0.185 | 0.129 | 0.156 |

**Table S4:** Results of multivariable-adjusted association of FRS and amyloid at baseline to predict cognition, using linear regression (Covariates: age, sex, *APOE4* genotype) in AD participants. There was no observed interaction at the baseline of FRS and Amyloid to predict cognition in the AD group.

*p-value<0.05, **p-value<0.01, ***p-value<0.001, ****p-value<0.0001

| **Association of Amyloid and Cognition in CN**  **Model: Cognition ~ Amyloid + covariates** | | | | | |
| --- | --- | --- | --- | --- | --- |
| **Diagnosis** | **Cognition** | **Predictors** | **Beta** | **SE** | **Adjusted p-value** |
| CN | MEM | Amyloid | -0.229 | 0.218 | 0.295 |
|  |  | Age | -0.022 | 0.003 | ****<0.0001 |
|  |  | Sex | 0.103 | 0.048 | 0.034 |
|  |  | APOE | -0.146 | 0.060 | 0.016 |
|  |  | Education | 0.046 | 0.005 | ***<0.0001 |
|  | EXF | Amyloid | -0.030 | 0.245 | 0.899 |
|  |  | Age | -0.035 | 0.003 | *****<0.0001 |
|  |  | Sex | 0.070 | 0.054 | 0.197 |
|  |  | APOE | -0.066 | 0.067 | 0.324 |
|  |  | Education | 0.057 | 0.006 | ****< 0.0001 |
|  | VSP | Amyloid | 0.329 | 0.456 | 0.470 |
|  |  | Age | -0.030 | 0.006 | ****<0.0001 |
|  |  | Sex | 0.060 | 0.101 | 0.554 |
|  |  | APOE | -0.261 | 0.123 | 0.035 |
|  |  | Education | 0.097 | 0.011 | ****<0.0001 |
|  | LAN | Amyloid | 0.382 | 0.246 | 0.122 |
|  |  | Age | -0.030 | 0.003 | ****<0.0001 |
|  |  | Sex | 0.162 | 0.054 | 0.003 |
|  |  | APOE | -0.042 | 0.067 | 0.532 |
|  |  | Education | 0.069 | 0.006 | ****<0.0001 |

**Table S5:** Results of multivariable-adjusted association of amyloid and cognition, using linear regression (Covariates: age, sex, *APOE4* genotype, and education) in CN participants.

*p-value<0.05, **p-value<0.01, ***p-value<0.001, ****p-value<0.0001

| **Association of Amyloid and Cognition in MCI**  **Model: Cognition ~ Amyloid + covariates** | | | | | |
| --- | --- | --- | --- | --- | --- |
| **Diagnosis** | **Cognition** | **Predictors** | **Beta** | **SE** | **Adjusted p-value** |
| MCI | MEM | Amyloid | -1.005 | 0.201 | ****<0.0001 |
|  |  | Age | -0.018 | 0.005 | ***<0.001 |
|  |  | Sex | -0.018 | 0.072 | 0.806 |
|  |  | APOE | -0.139 | 0.077 | 0.071 |
|  |  | Education | 0.024 | 0.008 | *<0.05 |
|  | EXF | Amyloid | -0.582 | 0.259 | 0.0956 |
|  |  | Age | -0.007 | 0.006 | 0.2335 |
|  |  | Sex | 0.160 | 0.091 | 0.081 |
|  |  | APOE | 0.059 | 0.097 | 0.542 |
|  |  | Education | 0.052 | 0.009 | ****<0.0001 |
|  | VSP | Amyloid | -0.655 | 0.507 | 0.198 |
|  |  | Age | -0.007 | 0.012 | 0.535 |
|  |  | Sex | -0.048 | 0.182 | 0.793 |
|  |  | APOE | -0.316 | 0.191 | 0.101 |
|  |  | Education | 0.119 | 0.019 | ****<0.0001 |
|  | LAN | Amyloid | -0.243 | 0.258 | 0.348 |
|  |  | Age | -0.019 | 0.006 | 0.10 |
|  |  | Sex | 0.151 | 0.092 | 0.104 |
|  |  | APOE | 0.121 | 0.098 | 0.221 |
|  |  | Education | 0.063 | 0.010 | ****<0.0001 |

**Table S6:** Results of multivariable-adjusted association of amyloid and cognition, using linear regression (Covariates: age, sex, *APOE4* genotype, and education) in MCI participants. Association of amyloid and memory was significant in MCI individuals, at baseline (β=-1.005, p-value < 0.001)

*p-value<0.05, **p-value<0.01, ***p-value<0.001, ****p-value<0.0001

| **Association of Amyloid and Cognition in AD**  **Model: Cognition ~ Amyloid + Covariates** | | | | | |
| --- | --- | --- | --- | --- | --- |
| **Diagnosis** | **Cognition** | **Predictors** | **Beta** | **SE** | **Adjusted p-value** |
| AD | MEM | Amyloid | -0.535 | 0.235 | 0.100 |
|  |  | Age | -0.002 | 0.006 | 0.708 |
|  |  | Sex | 0.006 | 0.110 | 0.955 |
|  |  | APOE | 0.126 | 0.090 | 0.163 |
|  |  | Education | 0.029 | 0.009 | *<0.05 |
|  | EXF | Amyloid | -0.341 | 0.385 | 0.379 |
|  |  | Age | 0.008 | 0.009 | 0.397 |
|  |  | Sex | 0.148 | 0.180 | 0.412 |
|  |  | APOE | 0.150 | 0.147 | 0.311 |
|  |  | Education | 0.038 | 0.156 | 0.0624 |
|  | VSP | Amyloid | -0.833 | 0.698 | 0.236 |
|  |  | Age | 0.038 | 0.017 | 0.119 |
|  |  | Sex | 0.519 | 0.333 | 0.123 |
|  |  | APOE | 0.061 | 0.267 | 0.820 |
|  |  | Education | 0.141 | 0.028 | ****<0.0001 |
|  | LAN | Amyloid | -0.470 | 0.332 | 0.161 |
|  |  | Age | 0.007 | 0.008 | 0.390 |
|  |  | Sex | 0.147 | 0.155 | 0.346 |
|  |  | APOE | 0.176 | 0.127 | 0.171 |
|  |  | Education | 0.063 | 0.013 | ****<0.0001 |

**Table S7:** Results of multivariable-adjusted association of amyloid and cognition using linear regression (Covariates: age, sex, *APOE4* genotype, and education) in AD participants.

*p-value<0.05, **p-value<0.01, ***p-value<0.001, ****p-value<0.0001

| **Dependent:**  **Cognition Variable(Y)** | **Model Predictor** | **Beta** | **SE** | **Adjusted p-value** |
| --- | --- | --- | --- | --- |
| Cognitively Normal (CN) | | | | |
| MEM (CN) | Amyloid*FRS*Time | 0.025 | 0.065 | 0.700 |
|  | Amyloid*Time | -0.193 | 0.058 | **<0.001 |
|  | FRS*Time | -0.004 | 0.013 | 0.731 |
|  | Amyloid * FRS | -0.039 | 0.235 | 0.867 |
|  | Amyloid | -0.032 | 0.215 | 0.881 |
|  | FRS | -0.028 | 0.048 | 0.558 |
|  | Time | 0.029 | 0.011 | **<0.010 |
|  | Age | -0.160 | 0.230 | ****<0.0001 |
|  | Sex | 0.188 | 0.049 | ****<0.0001 |
|  | APOE | -0.119 | 0.054 | *<0.05 |
|  | Education | 0.045 | 0.005 | ****<0.0001 |
| Mild Cognitive Impairment (MCI) | | | | |
| MEM (MCI) | Amyloid*FRS*Time | 0.015 | 0.055 | 0.778 |
|  | Amyloid*Time | -0.223 | 0.061 | ****<0.0001 |
|  | FRS*Time | -0.001 | 0.017 | 0.954 |
|  | Amyloid * FRS | 0.329 | 0.158 | *<0.05 |
|  | Amyloid | -0.992 | 0.208 | ****<0.0001 |
|  | FRS | -0.034 | 0.053 | 0.514 |
|  | Time | -0.013 | 0.021 | 0.555 |
|  | Age | -0.175 | 0.048 | ****<0.0001 |
|  | Sex | -0.040 | 0.082 | 0.629 |
|  | APOE | -0.161 | 0.078 | *<0.05 |
|  | Education | 0.022 | 0.008 | **<0.01 |
| Alzheimer’s Disease (AD) | | | | |
| MEM (AD) | Amyloid*FRS*Time | -0.077 | 0.158 | 0.626 |
|  | Amyloid*Time | 0.141 | 0.152 | 0.355 |
|  | FRS*Time | 0.098 | 0.069 | 0.153 |
|  | Amyloid * FRS | -0.067 | 0.236 | 0.777 |
|  | Amyloid | -0.383 | 0.247 | 0.125 |
|  | FRS | 0.057 | 0.108 | 0.6026 |
|  | Time | -0.252 | 0.077 | **<0.01 |
|  | Age | -0.019 | 0.048 | 0.683 |
|  | Sex | 0.015 | 0.123 | 0.903 |
|  | APOE | 0.127 | 0.086 | 0.144 |
|  | Education | 0.029 | 0.009 | **<0.01 |

**Table S8:** Results of linear mixed effects modeling of the synergistic interactive hypothesis of FRS and amyloid interaction on memory (MEM) domain over time in each diagnostic group.

Covariates: age, sex, *APOE4* genotype, and education

*p-value<0.05, **p-value<0.01, ***p-value<0.001, ****p-value<0.0001

| **Dependent:**  **Cognition Variable(Y)** | **Model Predictor** | **Beta** | **SE** | **Adjusted p-value** |
| --- | --- | --- | --- | --- |
| Cognitively Normal (CN) | | | | |
| EXF (CN) | Amyloid*FRS*Time | -0.041 | 0.076 | 0.588 |
|  | Amyloid*Time | -0.098 | 0.067 | 0.147 |
|  | FRS*Time | 0.001 | 0.015 | 0.921 |
|  | Amyloid * FRS | 0.047 | 0.283 | 0.867 |
|  | Amyloid | 0.062 | 0.259 | 0.812 |
|  | FRS | 0.006 | 0.058 | 0.920 |
|  | Time | 0.020 | 0.013 | 0.121 |
|  | Age | -0.243 | 0.028 | ****<0.0001 |
|  | Sex | 0.111 | 0.060 | 0.067 |
|  | APOE | -0.087 | 0.067 | 0.195 |
|  | Education | 0.056 | 0.006 | ****<0.0001 |
| Mild Cognitive Impairment (MCI) | | | | |
| EXF (MCI) | Amyloid*FRS*Time | 0.009 | 0.079 | 0.907 |
|  | Amyloid*Time | -0.345 | 0.088 | ***<0.001 |
|  | FRS*Time | 0.0004 | 0.025 | 0.988 |
|  | Amyloid * FRS | 0.095 | 0.217 | 0.662 |
|  | Amyloid | -0.342 | 0.282 | 0.228 |
|  | FRS | -0.052 | 0.072 | 0.470 |
|  | Time | 0.019 | 0.031 | 0.535 |
|  | Age | -0.040 | 0.064 | 0.531 |
|  | Sex | 0.222 | 0.108 | 0.042 |
|  | APOE | -0.041 | 0.103 | 0.692 |
|  | Education | 0.052 | 0.010 | ****<0.0001 |
| Alzheimer’s Disease (AD) | | | | |
| EXF (AD) | Amyloid*FRS*Time | 0.012 | 0.231 | 0.959 |
|  | Amyloid*Time | -0.094 | 0.230 | 0.684 |
|  | FRS*Time | 0.046 | 0.099 | 0.644 |
|  | Amyloid * FRS | -0.216 | 0.390 | 0.583 |
|  | Amyloid | -0.378 | 0.406 | 0.354 |
|  | FRS | 0.035 | 0.178 | 0.844 |
|  | Time | -0.144 | 0.115 | 0.208 |
|  | Age | 0.105 | 0.075 | 0.169 |
|  | Sex | 0.309 | 0.190 | 0.108 |
|  | APOE | 0.060 | 0.133 | 0.651 |
|  | Education | 0.050 | 0.014 | ****<0.0001 |

**Table S9:** Results of linear mixed effects modeling of the synergistic hypothesis of FRS and amyloid interaction on Executive functioning (EXF) over time, in each diagnosis group.

Covariates: age, sex, *APOE4* genotype and education.

*p-value<0.05, **p-value<0.01, ***p-value<0.001, ****p-value<0.0001

| **Dependent:**  **Cognition Variable(Y)** | **Model Predictor** | **Beta** | **SE** | **Adjusted p_value** |
| --- | --- | --- | --- | --- |
| Cognitively Normal (CN) | | | | |
| VSP (CN) | Amyloid*FRS*Time | 0.014 | 0.172 | 0.935 |
|  | Amyloid*Time | -0.370 | 0.152 | *<0.05 |
|  | FRS*Time | -0.009 | 0.034 | 0.798 |
|  | Amyloid * FRS | 0.577 | 0.515 | 0.263 |
|  | Amyloid | 0.529 | 0.468 | 0.259 |
|  | FRS | -0.113 | 0.103 | 0.276 |
|  | Time | -0.031 | 0.030 | 0.295 |
|  | Age | 0.234 | 0.0472 | ****<0.0001 |
|  | Sex | 0.033 | 0.101 | 0.741 |
|  | APOE | -0.173 | 0.111 | 0.121 |
|  | Education | 0.099 | 0.009 | ****<0.0001 |
| Mild Cognitive Impairment (MCI) | | | | |
| VSP(MCI) | Amyloid*FRS*Time | 0.327 | 0.162 | *<0.05 |
|  | Amyloid*Time | -0.837 | 0.177 | ***<0.0001 |
|  | FRS*Time | -0.077 | 0.054 | 0.148 |
|  | Amyloid * FRS | -0.031 | 0.402 | 0.938 |
|  | Amyloid | -0.512 | 0.517 | 0.324 |
|  | FRS | -0.133 | 0.134 | 0.320 |
|  | Time | 0.063 | 0.061 | 0.303 |
|  | Age | 0.046 | 0.112 | 0.679 |
|  | Sex | 0.033 | 0.193 | 0.864 |
|  | APOE | -0.302 | 0.181 | 0.098 |
|  | Education | 0.111 | 0.018 | ****<0.0001 |
| Alzheimer’s Disease (AD) | | | | |
| VSP (AD) | Amyloid*FRS*Time | -0.407 | 0.396 | 0.304 |
|  | Amyloid*Time | 0.117 | 0.388 | 0.764 |
|  | FRS*Time | 0.238 | 0.173 | 0.168 |
|  | Amyloid * FRS | -0.391 | 0.745 | 0.601 |
|  | Amyloid | -0.785 | 0.750 | 0.298 |
|  | FRS | 0.116 | 0.347 | 0.738 |
|  | Time | -0.425 | 0.187 | 0.092 |
|  | Age | 0.347 | 0.154 | 0.108 |
|  | Sex | 0.564 | 0.379 | 0.141 |
|  | APOE | -0.117 | 0.263 | 0.657 |
|  | Education | 0.121 | 0.027 | ****<0.0001 |

**Table S10:** Results of linear mixed effects modeling of the synergistic hypothesis of FRS and amyloid interaction on visuospatial functioning (VSP) over time, in each diagnosis group.

Covariates: age, sex, *APOE4* genotype and education.

*p-value<0.05, **p-value<0.01, ***p-value<0.001, ****p-value<0.0001

| **Dependent:**  **Cognition Variable(Y)** | **Model Predictor** | **Beta** | **SE** | **Adjusted p_value** |
| --- | --- | --- | --- | --- |
| **Language Domain** | | | | |
| Cognitively Normal (CN) | | | | |
| LAN (CN) | Amyloid*FRS*Time | 0.041 | 0.051 | 0.419 |
|  | Amyloid*Time | -0.121 | 0.045 | *<0.05 |
|  | FRS*Time | -0.014 | 0.010 | 0.156 |
|  | Amyloid * FRS | 0.238 | 0.281 | 0.398 |
|  | Amyloid | 0.539 | 0.256 | 0.144 |
|  | FRS | -0.031 | 0.058 | 0.592 |
|  | Time | 0.020 | 0.009 | 0.108 |
|  | Age | -0.221 | 0.028 | ****<0.0001 |
|  | Sex | 0.191 | 0.060 | ***<0.001 |
|  | APOE | 0.072 | 0.066 | 0.275 |
|  | Education | 0.069 | 0.006 | ****<0.0001 |
| Mild Cognitive Impairment (MCI) | | | | |
| LAN(MCI) | Amyloid*FRS*Time | 0.073 | 0.064 | 0.254 |
|  | Amyloid*Time | -0.387 | 0.071 | ***<0.0001 |
|  | FRS*Time | -0.016 | 0.020 | 0.414 |
|  | Amyloid * FRS | 0.203 | 0.200 | 0.313 |
|  | Amyloid | -0.296 | 0.261 | 0.259 |
|  | FRS | -0.060 | 0.067 | 0.372 |
|  | Time | 0.015 | 0.025 | 0.540 |
|  | Age | -0.124 | 0.060 | 0.164 |
|  | Sex | 0.167 | 0.103 | 0.105 |
|  | APOE | 0.105 | 0.097 | 0.281 |
|  | Education | 0.060 | 0.010 | ****<0.0001 |
| Alzheimer’s Disease (AD) | | | | |
| LAN(AD) | Amyloid*FRS*Time | 0.066 | 0.150 | 0.661 |
|  | Amyloid*Time | -0.170 | 0.145 | 0.241 |
|  | FRS*Time | 0.018 | 0.065 | 0.778 |
|  | Amyloid * FRS | -0.265 | 0.357 | 0.460 |
|  | Amyloid | -0.396 | 0.375 | 0.293 |
|  | FRS | 0.079 | 0.164 | 0.632 |
|  | Time | -0.180 | 0.074 | 0.056 |
|  | Age | 0.035 | 0.074 | 0.638 |
|  | Sex | 0.185 | 0.190 | 0.333 |
|  | APOE | 0.221 | 0.134 | 0.104 |
|  | Education | 0.059 | 0.014 | ****<0.0001 |

**Table S11:** Results of linear mixed effects modeling of the synergistic hypothesis of the interactive contribution of FRS and amyloid on language (LAN) performance over time, in each diagnosis group.

Covariates: age, sex, *APOE4* genotype and education.

*p-value<0.05, **p-value<0.01, ***p-value<0.001, ****p-value<0.0001

| **Dependent:**  **Cognition Variable(Y)** | **Model Predictors** | **Beta** | **SE** | **Adjusted p-value** |
| --- | --- | --- | --- | --- |
| Cognitively Normal (CN) | | | | |
| MEM (CN) | FRS*Time | 0.000 | 0.007 | 0.966 |
|  | Amyloid*Time | -0.199 | 0.056 | ****<0.0001 |
|  | FRS | -0.035 | 0.030 | 0.244 |
|  | Amyloid | 0.024 | 0.209 | 0.909 |
|  | Time | 0.030 | 0.111 | **<0.01 |
|  | Age | -0.160 | 0.023 | ****<0.0001 |
|  | Sex | 0.188 | 0.049 | ****<0.0001 |
|  | APOE | -0.120 | 0.053 | *<0.05 |
|  | Education | 0.045 | 0.005 | ****<0.0001 |
| Mild Cognitive Impairment (MCI) | | | | |
| MEM (MCI) | FRS*Time | 0.003 | 0.010 | 0.766 |
|  | Amyloid*Time | -0.219 | 0.059 | ****<0.0001 |
|  | FRS | 0.047 | 0.036 | 0.190 |
|  | Amyloid | -0.916 | 0.207 | ****<0.0001 |
|  | Time | -0.014 | 0.021 | 0.512 |
|  | Age | -0.168 | 0.048 | ***<0.001 |
|  | Sex | -0.036 | 0.083 | 0.663 |
|  | APOE | -0.152 | 0.078 | *<0.05 |
|  | Education | 0.023 | 0.008 | **<0.01 |
| Alzheimer’s Disease (AD) | | | | |
| MEM (AD) | FRS*Time | 0.067 | 0.028 | **<0.01 |
|  | Amyloid*Time | 0.118 | 0.144 | 0.4170 |
|  | FRS | 0.031 | 0.057 | 0.585 |
|  | Amyloid | -0.399 | 0.238 | 0.097 |
|  | Time | -0.263 | 0.071 | ***<0.001 |
|  | Age | -0.021 | 0.047 | 0.653 |
|  | Sex | 0.014 | 0.122 | 0.907 |
|  | APOE | 0.124 | 0.085 | 0.150 |
|  | Education | 0.030 | 0.009 | ***<0.001 |

**Table S12:** Results of linear mixed effects modeling of the additive hypothesis of the independent contribution of FRS and amyloid on memory (MEM) domain over time, in each diagnostic group.

Covariates: age, sex, *APOE4* genotype and education

*p-value<0.05, **p-value<0.01, ***p-value<0.001, ****p-value<0.0001

| **Dependent:**  **Cognition Variable(Y)** | **Model Predictors** | **Beta** | **SE** | **Adjusted p_value** |
| --- | --- | --- | --- | --- |
| Cognitively Normal (CN) | | | | |
| EXF (CN) | FRS*Time | -0.005 | 0.008 | 0.535 |
|  | Amyloid*Time | -0.089 | 0.065 | 0.176 |
|  | FRS | 0.013 | 0.036 | 0.709 |
|  | Amyloid | 0.052 | 0.252 | 0.837 |
|  | Time | 0.019 | 0.013 | 0.140 |
|  | Age | -0.243 | 0.028 | ****<0.0001 |
|  | Sex | 0.111 | 0.060 | 0.066 |
|  | APOE | -0.086 | 0.066 | 0.190 |
|  | Education | 0.056 | 0.006 | ****<0.0001 |
| Mild Cognitive Impairment (MCI) | | | | |
| EXF (MCI) | FRS*Time | 0.003 | 0.015 | 0.855 |
|  | Amyloid*Time | -0.342 | 0.085 | ***<0.001 |
|  | FRS | -0.029 | 0.048 | 0.550 |
|  | Amyloid | -0.319 | 0.274 | 0.250 |
|  | Time | 0.018 | 0.030 | 0.541 |
|  | Age | -0.038 | 0.063 | 0.549 |
|  | Sex | 0.223 | 0.108 | 0.040 |
|  | APOE | -0.038 | 0.102 | 0.707 |
|  | Education | 0.052 | 0.010 | ****<0.0001 |
| Alzheimer’s Disease (AD) | | | | |
| EXF (AD) | FRS*Time | 0.051 | 0.041 | 0.210 |
|  | Amyloid*Time | 0.088 | 0.220 | 0.691 |
|  | FRS | -0.048 | 0.092 | 0.601 |
|  | Amyloid | -0.433 | 0.391 | 0.271 |
|  | Time | -0.147 | 0.107 | 0.170 |
|  | Age | 0.102 | 0.075 | 0.178 |
|  | Sex | 0.309 | 0.189 | 0.107 |
|  | APOE | 0.052 | 0.132 | 0.695 |
|  | Education | 0.051 | 0.014 | ****<0.0001 |

**Table S13:** Results of linear mixed effects modeling of the additive hypothesis of the independent contribution of FRS and amyloid on Executive functioning (EXF) over time, in each diagnosis group.

Covariates: age, sex, *APOE4* genotype and education.

*p-value<0.05, **p-value<0.01, ***p-value<0.001, ****p-value<0.0001

| **Dependent:**  **Cognition Variable(Y)** | **Model Predictors** | **Beta** | **SE** | **Adjusted p_value** |
| --- | --- | --- | --- | --- |
| Cognitively Normal (CN) | | | | |
| VSP (CN) | FRS*Time | -0.006 | 0.019 | 0.769 |
|  | Amyloid*Time | -0.375 | 0.149 | *<0.05 |
|  | FRS | -0.020 | 0.063 | 0.745 |
|  | Amyloid | 0.423 | 0.460 | 0.359 |
|  | Time | -0.031 | 0.030 | 0.300 |
|  | Age | 0.241 | 0.047 | ****<0.0001 |
|  | Sex | 0.038 | 0.101 | 0.708 |
|  | APOE | -0.148 | 0.110 | 0.178 |
|  | Education | 0.100 | 0.009 | ****<0.0001 |
| Mild Cognitive Impairment (MCI) | | | | |
| VSP (MCI) | FRS*Time | 0.012 | 0.031 | 0.706 |
|  | Amyloid*Time | -0.741 | 0.174 | ***<0.0001 |
|  | FRS | -0.141 | 0.087 | 0.109 |
|  | Amyloid | -0.532 | 0.505 | 0.294 |
|  | Time | 0.040 | 0.061 | 0.513 |
|  | Age | -0.043 | 0.111 | 0.697 |
|  | Sex | 0.027 | 0.192 | 0.887 |
|  | APOE | -0.296 | 0.179 | 0.101 |
|  | Education | 0.112 | 0.018 | ****<0.0001 |
| Alzheimer’s Disease (AD) | | | | |
| VSP (AD) | FRS*Time | 0.076 | 0.071 | 0.282 |
|  | Amyloid*Time | 0.067 | 0.387 | 0.864 |
|  | FRS | -0.042 | 0.176 | 0.812 |
|  | Amyloid | -0.864 | 0.729 | 0.240 |
|  | Time | -0.375 | 0.182 | 0.156 |
|  | Age | 0.342 | 0.152 | 0.112 |
|  | Sex | 0.576 | 0.375 | 0.129 |
|  | APOE | -0.135 | 0.260 | 0.604 |
|  | Education | 0.122 | 0.027 | ****<0.0001 |

**Table S14:** Results of linear mixed effects modeling of the additive hypothesis of the independent contribution of FRS and amyloid on visuospatial functioning (VSP) over time, in each diagnosis group.

Covariates: age, sex, *APOE4* genotype and education.

*p-value<0.05, **p-value<0.01, ***p-value<0.001, ****p-value<0.0001

| **Dependent:**  **Cognition Variable(Y)** | **Model Predictors** | **Beta** | **SE** | **Adjusted p-value** |
| --- | --- | --- | --- | --- |
| Cognitively Normal (CN) | | | | |
| LAN (CN) | FRS*Time | -0.007 | 0.006 | 0.182 |
|  | Amyloid*Time | 0.131 | 0.044 | *<0.05 |
|  | FRS | 0.008 | 0.036 | 0.831 |
|  | Amyloid | 0.491 | 0.250 | 0.200 |
|  | Time | 0.021 | 0.009 | 0.056 |
|  | Age | -0.225 | 0.028 | ****<0.0001 |
|  | Sex | 0.193 | 0.060 | **<0.01 |
|  | APOE | -0.061 | 0.065 | 0.350 |
|  | Education | 0.070 | 0.006 | ****<0.0001 |
| Mild Cognitive Impairment (MCI) | | | | |
| LAN (MCI) | FRS*Time | 0.002 | 0.012 | 0.864 |
|  | Amyloid*Time | -0.366 | 0.068 | ***<0.0001 |
|  | FRS | -0.010 | 0.045 | 0.830 |
|  | Amyloid | -0.254 | 0.257 | 0.326 |
|  | Time | 0.010 | 0.0246 | 0.692 |
|  | Age | 0.119 | 0.060 | 0.196 |
|  | Sex | 0.171 | 0.103 | 0.098 |
|  | APOE | 0.112 | 0.097 | 0.251 |
|  | Education | 0.060 | 0.010 | ***<0.0001 |
| Alzheimer’s Disease (AD) | | | | |
| LAN (AD) | FRS*Time | 0.044 | 0.026 | 0.093 |
|  | Amyloid*Time | 0.152 | 0.138 | 0.270 |
|  | FRS | -0.025 | 0.088 | 0.778 |
|  | Amyloid | -0.463 | 0.362 | 0.204 |
|  | Time | -0.191 | 0.068 | *<0.05 |
|  | Age | 0.031 | 0.074 | 0.673 |
|  | Sex | 0.188 | 0.189 | 0.322 |
|  | APOE | 0.210 | 0.133 | 0.119 |
|  | Education | 0.060 | 0.014 | ****<0.0001 |

**Table S15:** Results of linear mixed effects modeling of the additive hypothesis of the independent contributions of FRS and amyloid on language (LAN) performance over time, in each diagnosis group.

Covariates: age, sex, *APOE4* genotype and education.

*p-value<0.05, **p-value<0.01, ***p-value<0.001, ****p-value<0.0001

**SUPPLEMENTARY TEXT**

**Harmonization of cognitive domains from the Korean Brain Aging Study for Early Diagnosis and Prediction of Alzheimer’s Disease (KBASE)**

**S-E. Choi*, P. Scollard*, M. L. Lee*, B. Klinedinst*, S. Mukherjee, L. E. Gibbons, C. Nakano, E. Trittschuh, J. Mez, A. J. Saykin, K. Nho, M. Cuccaro, S. Turner, L. Dumitrescu, T. J. Hohman, D. Yi, P. K. Crane for the Alzheimer’s Disease Sequencing Project Phenotype Harmonization Consortium**

*** These authors contributed equally.**

**Introduction:** The Korean Brain Aging Study for Early Diagnosis and Prediction of Alzheimer’s Disease (KBASE) is a study of older adults set in South Korea. It includes multiple modalities including imaging, cognition, and genetics. The Cognition Core of the Alzheimer’s Disease Sequencing Project Phenotype Harmonization Consortium (ADSP-PHC) worked on KBASE in 2023 to harmonize and co-calibrate cognitive scores together with our growing item bank of North American-based studies. This document provides an overview of the workflow.

**General considerations:** This is our first study to co-calibrate cognitive domains among participants whose cognition was assessed in Korean. The neuropsychological battery was designed by a bilingual Korean- English neuropsychologist (Dr. Yi). Each cognitive item in the KBASE neuropsychological battery was one we had encountered before in English. We carefully considered the question of whether the stimulus and response formats were sufficiently similar for English and Korean administrations. Where we determined these to be sufficiently similar, we could use English language derived item parameters; these items served to anchor the English and Korean administrations (hence “anchor items”). We freely estimated parameters for the non-anchor items, i.e. items that we determined were too dissimilar between the English and Korean testing contexts. We also note that the study design of KBASE was such that not all the tests were administered at all visits.

In our discussions regarding the testing context, we learned some important considerations about cohorts and sex differences in education. In particular, for older women in this study, women may have had substantially less educational attainment than men of similar ages. There may also be important cultural and gender differences in terms of willingness to guess or attempt an item. We were not able to assess these differences with the study design, however, analyses that use these scores in conjunction with other English-speaking cohorts should take the potential impact of cultural differences into consideration.

Based on our meetings with Dr. Yi and our expert panel of Dr. Trittschuh, Dr. Mez, and Dr. Saykin, our item analyses implemented the following protocol for anchoring. First, if there was an item administered in KBASE in a way that differed from the English administration, then it was deemed a non-anchor (KBASE-unique non-anchors). Second, an item is also a non-anchor if language or cultural differences were considered likely to lead to a difference in the measurement properties of the items (Korean vs. English context non-anchors). Anchor items were those that were identical between KBASE and studies we had previously analyzed, and where the Korean vs. English context were thought to be similar enough to anchor. Composite scores for each of the domains were derived using the same workflow as mentioned in our cognitive harmonization paper.^1^ In brief, scores were estimated for all individuals and visits using a separate model for each domain. The models included the same secondary factors as were used at co-calibration. All item parameters were fixed at previously estimated values, either from our item bank or co-calibration step.

**The Memory Domain:**

**Items:** Thirteen items were assigned to this domain which include CERAD word list trials (3 trials) along with delayed recall and recognition, logical memory immediate and delayed recall for two stories followed by logical memory recognition, CERAD constructional praxis delayed recall, and a 3- and 30-minutes delayed recall from the Rey complex figure test (2 items). There are seven anchors and six non-anchors. An overview of the items and their anchor status can be found in Table 1.

**Pre-calibration**

The protocol to prepare data for this phase resulted in high missingness among logical memory items which are all non-anchors. This was due to the study design as mentioned above. We solved this issue by keeping any visit with logical memory recognition before we took the last available visits. We used agglomerative hierarchical clustering to generate a heatmap for the memory items. Based on that, we tested several models that included a simple single factor model followed by three different bifactor models. Our chosen model was a bifactor model with secondary structures for CERAD wordlist trials 1 to 3 and CERAD wordlist delayed recall, and a second secondary factor with the constructional praxis delayed recall and the two Rey complex figure delayed recall items.

**Co-calibration**

We used the same sample for this step as laid out under co-calibration. The same sample restriction of keeping any visit with logical memory recognition was also forced in this step. We fixed item parameters for all the seven anchors, while item parameters for six non-anchor were estimated.

**Table 1.** Cognitive items for the memory domain.

| **Raw variable** | **Recoded variable** | **Description** | **Anchor** | **Secondary structure** |
| --- | --- | --- | --- | --- |
| j4_1st | rwordt1 | CERAD word list trial 1 | Yes | F10 |
| j4_2nd | rwordt2 | CERAD word list trial 2 | Yes | F10 |
| j4_3rd | rwordt3 | CERAD word list trial 3 | Yes | F10 |
| j6 | rrecall | CEARD word list delayed recall | Yes | F10 |
| j7 | rj7 | CERAD word list recognition | No |  |
| j8 | rcprecll | CERAD constructional praxis delayed recall | Yes |  |
| l3_rcft_3min | rrcft_3m | Rey Complex Figure Test 3 min delayed recall | Yes | F27 |
| l8_rcft_30min | rrcft_30 | Rey Complex Figure Test 30 min delayed recall | Yes | F27 |
| l4_a | rl4_a | WMS-IV K logical memory, immediate story A | No | F29 |
| l4_b | rl4_b | WMS-IV K logical memory, immediate story B | No | F30 |
| l10_a | rl10_a | WMS-IV K logical memory, delayed story A | No | F29 |
| l10_b | rl10_b | WMS-IV K logical memory, delayed story B | No | F30 |
| l10_recog | rl10recg | WMS-IV K logical memory, recognition | No |  |

**The Executive Functioning Domain:**

**Items:** There were six items in Executive Functioning: clock drawing scored on a 15-point scale, Stroop color-word, WAIS-R digit span backwards and forwards, and trails A and B. For executive functioning items, it was determined that all KBASE items except the clock drawing item could be anchored to the English item bank. An overview of the items and their anchor status can be found in Table 2.

**Pre-calibration:** We used agglomerative hierarchical clustering to generate a heatmap for the executive functioning items. Two pre-calibration models were run: a single factor model and a bifactor model, which included a secondary factor for the two digit span items and an additional factor for the two trail making items. In order to remain consistent with our item bank, we decided to move forward with the bifactor model.

**Co-calibration:** Additional sample restrictions were required in order to obtain a sample with low missingness across items. The pre- and co-calibration sample was restricted to:

- Visits with Stroop and/or clock drawing (these seemed to follow the same pattern of administration).
- Visits with at least one of trails A, trails B, digit span forwards, or digit span backwards.
- Visits where the individual was at least 60.
- From this pool of visits, we selected the most recent for each individual.

The only non-anchor item was clock drawing. This item was estimated at the co-calibration stage.

**Table 2.** Cognitive items for the executive functioning domain.

| **Raw Variable** | **Recoded**  **Variable** | **Description** | **Anchor** | **Secondary structure** |
| --- | --- | --- | --- | --- |
| k3_1 | rclk15k | Clock drawing, 1:45, 15-point scale | No |  |
| k1_cw | rstpcw | Stroop color-word (Golden) | Yes |  |
| ds_forwardl12 | rdigfor | WAIS-R Digit span forwards | Yes | F1 |
| ds_backwardl12 | rdigbac | WAIS-R Digit span backwrds | Yes | F1 |
| tmt_al11 | rtrailsa | Trail making A | Yes | F7 |
| Tmt_bl11 | rtrailsb | Trail making B | Yes | F7 |

**The Language Domain:**

**Items:** The seven language items available (based on their English names) were Animal Naming, Stroop Words and Stroop Colors, the Boston Naming Test (BNT), and phonemic fluency for the letters F, A, and S. An overview of the items and their anchor status can be found in Table 3.

**Pre-calibration**: We used agglomerative hierarchical clustering to generate a heatmap for the seven items. Three potential secondary structures were noted and evaluated: (a) correlations between the Stroop items; (b) correlations between the phonemic fluency items; and (c) a correlation between animal fluency and BNT. Structures (a) and (b) were observed in prior studies with English as the testing language. We opted to skip a potential structure for (c) given that it had not been observed in English language data sets and produced the smallest pairwise correlation. A significant model improvement was observed for the model when including the F/A/S items as a secondary factor compared to a single factor model. A smaller improvement was noted if the Stroop structure was also included. We decided to move forward with a bifactor model incorporating the F/A/S structure.

**Co-calibration:** Sample restrictions were required to obtain a co-calibration sample with low missingness across items:

- Visits with at least one phonemic fluency item
- Visits where the participant was least 60 years old
- From this pool of visits, we selected the most recent visit from each participant

The three phonemic fluency items, Stroop Words, and the Boston Naming Test were estimated at this stage.

**Table 3.** Cognitive items for the language domain.

| **Raw Variable** | **Recoded**  **Variable** | **Description** | **Anchor** | **Secondary structure** |
| --- | --- | --- | --- | --- |
| k1_w | rk1_w | Stroop Words | No |  |
| k1_c | rstpc | Stroop Colors | Yes |  |
| j1_tot | rcatanim | Animal Fluency | Yes |  |
| j2_tot | rj2_tot | Boston Naming Test | No |  |
| l2_1 | rl2_1 | Phonemic Fluency - F | No | F2 |
| l2_2 | rl2_2 | Phonemic Fluency - A | No | F2 |
| l2_3 | rl2_3 | Phonemic Fluency - S | No | F2 |

**The Visuospatial functioning Domain:**

**Items:** Four items were assigned to this domain: CERAD constructional Praxis, Rey complex figure task, Clock Drawing task, and Block design task. The CERAD and the Rey items were identified as anchors in our item bank. Two items (Rey Copy and Block Design) were administered every other visit, leading to roughly 50% missingness in the dataset. We considered different sample restrictions on the dataset and optimized the sample for including these two items. Visits included in the harmonization had to contain both items.

**Pre-calibration:**

A single factor model was run. Bifactor models were not considered because there are too few items and the correlations are not strong.

**Co-calibration:**

We restricted the sample to exclude visits that did not have the Rey and Block design items.

Sample restriction steps:

- Visits with Rey and Block design
- Visits where participants were age 60 or over
- Most recent visit for each individual

The item parameters for non-anchor items were estimated at the co-calibration stage.

**Table 4.** Cognitive items for the visuospatial functioning domain.

| Raw Variable | Recoded Variable | Description | Anchor | Secondary Structure |
| --- | --- | --- | --- | --- |
| j5 | rcpintot | Cerad Constructional Praxis | Yes |  |
| l1_rcft_copy | rreycto | Rey Complex Figure | Yes |  |
| k3_2 | rclockdr | Clock Drawing | No |  |
| l5 | rblkdsn | Block Design | No |  |

**References:**

1. Mukherjee S, Choi SE, Lee ML, et al. Cognitive domain harmonization and cocalibration in studies of older adults. *Neuropsychology*. Aug 4 2022:No Pagination Specified-No Pagination Specified. doi:10.1037/neu0000835
